# Outcomes of school-aged children following traumatic brain injury from road traffic accidents in Malaysia: A retrospective study

**DOI:** 10.1371/journal.pone.0351136

**Published:** 2026-06-16

**Authors:** Esther Jimbih, Mazlina Mazlan, Ria Waran, Vicknes Waran

**Affiliations:** 1 Department of Rehabilitation Medicine, Faculty of Medicine, Universiti Malaya, Kuala Lumpur, Malaysia; 2 NeuroSphere Sdn. Bhd., Selangor, Malaysia; 3 Subang Jaya Medical Centre, Selangor, Malaysia; Universiti Sains Malaysia, MALAYSIA

## Abstract

**Background:**

Traumatic brain injury (TBI) resulting from road traffic accidents (RTAs) is a leading cause of morbidity in children globally. However, paediatric TBI outcomes are understudied in Southeast Asia.

**Objective:**

To examine functional outcomes and associated factors among school-aged children with RTA-related TBI in Malaysia.

**Methods:**

This retrospective study analysed medical records of 542 children aged 7–17 years with first-time TBI from RTAs. Data on sociodemographics, injury characteristics, complications, and outcomes were collected. The subjects’ reports were grouped into three categories for analyses: young children (7–9-year-olds), adolescents (10–14-year-olds) and older adolescents (15–17-year-olds). Functional status was assessed using the Glasgow Outcome Scale–Extended for Paediatrics (GOS-E Peds), and reported as good (category 1 and 2) or poor (category 3–8) outcomes. Descriptive statistics and multivariate logistic regression were used.

**Results:**

The majority of children were male (81.5%) with a mean age of 14.3 years. Motorcycles were the predominant accident mode (76.0%) especially among adolescents and older adolescents. The mean duration between the RTAs and report review was 20.7 ± 14.9 months. Only 27.5% of the children achieved good functional outcomes. Young children had the highest rate of recovery (34.5%), though not statistically significant. Among older adolescents, mild TBI significantly increased the odds of a good outcome (OR=2.5; 95% CI: 1.29–4.83; *p* = 0.007), while tracheostomy was strongly associated with poor outcomes (OR=7.02; 95% CI: 2.06–23.93; *p* = 0.002). Despite high rate of return to education, a significant proportion of children faced academic challenges post-brain injury.

**Conclusion:**

Motorcycles remain the primary mode of RTA-related TBI among Malaysian children, particularly among adolescents. There was no significant difference in outcomes between the three age groups, although the young children had the highest rate of recovery. A substantial proportion of the children experience ongoing functional and academic difficulties despite returning to school. This highlights the need for coordinated clinical, educational, and policy responses in settings with limited paediatric rehabilitation infrastructure.

## Introduction

Traumatic brain injury (TBI) remains a leading cause of disability and mortality among children worldwide. Annually, it affects more than three million children, with road traffic accidents (RTAs) and falls being the predominant contributors and common causes of injury-related death and disability in low- and middle- income countries (LMICs). [1,2] RTAs represent a significant public health concern in LMICs including Malaysia. An epidemiological study in the central region of west Malaysia reported that 12.7% of paediatric trauma patients sustained brain injuries, with 95.4% of those cases attributed to RTAs. [3] Furthermore, motorcycle accidents accounted for 80.2% of the RTAs causing brain injuries. Notably, more than two-thirds of the TBI cases occurred in older adolescents aged 15–19 years, who were predominantly male. [3]

Although road safety measures have improved, children remain vulnerable to injuries due to their physical fragility and limited traffic awareness. It has been suggested that children respond to and recover from brain injuries differently than adults. This is largely attributed to the greater plasticity of their developing brains. [4,5] Younger patients often exhibit continued improvements following brain injury. However, recovery outcomes still vary significantly depending on age and injury severity. Very young children under six years of age generally experience poorer functional recovery compared to early adolescents and adolescents. [6–8] This discrepancy is likely due to ongoing brain development and maturation processes during early childhood.

Despite increasing recognition of age-related differences in paediatric TBI recovery, research in Southeast Asia has largely focused on adult populations, with limited attention given to children. [9–11] Local studies looking at TBI outcomes in children were conducted more than a decade ago, and to the authors’ knowledge, there have been no other published studies with Malaysian subjects since that time. [12–14] To address this gap, the present study examines outcomes among school-aged children in Malaysia who have sustained TBI as a result of RTA. School-aged children are in a crucial phase of cognitive, social and emotional development, thus TBI at this stage may result in long-term deficits that affect education and life trajectory. This study aims to examine the functional outcomes and the factors influencing a good functional outcome among school-aged children across three different age groups.

## Methods

This study used a retrospective cohort design and was approved by the Universiti Malaya Medical Centre Medical Research Ethics Committee (MREC-202517–14567). It involved the examination of specialist medical reports on paediatric TBI cases resulting from RTAs in Malaysia over a ten-year period (2009–2019). Each subject had one medical report prepared by a senior medical consultant, who received referrals from across Malaysia. These reports were generated for insurance claim purposes, and there were no duplicate reports per subject. Data were accessed for research purposes on 17 February, 2025. All data were fully anonymised immediately after access and before analysis. As this was a retrospective study of medical records, the ethics committee waived the requirement for informed consent. This study extends a previous study which was focused on an adult population dataset. [15]

Inclusion criteria were children aged 7–17 years (the Malaysian school-going age) at the time of injury, who sustained TBI due to RTAs, and had no prior TBIs. Patients with a prior brain injuries or pre-existing medical conditions causing significant cognitive, behavioural, or physical disabilities were excluded. The following information was gathered from the specialist medical reports which included sociodemographic data, injury details, complications, and functional outcomes at home and in the community. Additional relevant information gathered were complaints reported at the time of review and academic challenges encountered by children who had returned to school following the injury.

Sociodemographic data included age at the time of injury, time since the injury, gender, ethnicity, pre- and post-injury education and possible employment status. Injury details included accident location, mode, TBI severity, neurosurgical interventions, presence of concomitant injuries and associated interventions, intensive care unit (ICU) admission, presence of acute medical issues and tracheostomy. Post-TBI complications included post-traumatic headaches, pain, seizures and behavioural problems. Medication information included whether subjects were prescribed anti-epileptic drugs.

The time since injury was defined as the time of the injury to the specialist review and categorized into: less than two years and two years or more. Pre-injury education status was classified into three categories: neither studying nor working, studying, or working. Accident locations were grouped based on regions in west Malaysia: northern, east coast, central, and southern. [16] There were no reports from east Malaysia.

The mode of accident was classified into five categories: car, motorcycle, lorry, pedestrian, and others. The severity of TBI was determined based on the Glasgow Coma Scale (GCS) score recorded at hospital presentation: scores of 13–15 indicated mild TBI, 9–12 moderate TBI, and 8 or below severe TBI. Concomitant injuries include all other injuries involving neck, facial, chest, abdominal, spine, upper extremities, lower extremities and external injury. Acute medical issues during admission included, but were not limited to, pneumonia, sepsis, and surgical site infections. Behavioural problems were categorised as: easily angered, withdrawn, developmentally inappropriate, mixed (exhibit a combined clinical presentation), or none.

Functional outcomes were assessed using available information in specialist medical reports, with the duration post-injury varying across cases. The Glasgow Outcome Scale–Extended for Paediatrics (GOS-E Peds) was used to determine functional status. [17] This validated tool is an age-appropriate adaptation of the Glasgow Outcome Scale–Extended (GOS-E). While the GOS-E Peds was developed for children up to 16 years of age, we applied it to our entire school-aged cohort, including participants over 16, to ensure consistent measurement. The authors agree that this is appropriate and clinically meaningful as the core functional domains of school reintegration, academic performance, and peer relationships remain the most relevant indicators of outcome for this population. This approach may differ from studies using the standard GOS-E for older adolescents and might be less sensitive to deficits in more complex, adult-oriented functional tasks such as return to work, ability to manage finances and independence in running a household.

The GOS-E Peds categorizes outcomes into eight levels: upper good recovery (category 1), lower good recovery (category 2), upper moderate disability (category 3), lower moderate disability (category 4), upper severe disability (category 5), lower severe disability (category 6), vegetative state (category 7), and death (category 8). Two researchers independently scored each patient using the structured GOS-E Peds form to ensure standardization. Given the wide developmental span in this population, outcome interpretation was contextualised within age-specific groups to better reflect functional variation across stages of childhood and adolescence.

### Statistical analysis

Data were analysed using IBM® SPSS® Statistics, Version 27 (Armonk, NY). To reflect developmental and clinical differences across the paediatric age spectrum, patients were grouped into three categories for analyses: young children (7–9-year-olds), adolescents (10–14-year-olds) and older adolescents (15–17-year-olds). This subgrouping allowed us to explore whether clinical profiles, injury characteristics, or functional outcomes varied meaningfully across age groups, which aligned with the exploratory nature of the study. Each group was analysed separately to assess sociodemographic characteristics, functional outcomes, and common complaints post-TBI. Descriptive statistics were used to summarise sociodemographic information, clinical characteristics, and common complaints. Continuous data were reported as means with standard deviations, while categorical data were presented as frequencies and percentages.

In this study, a ‘good’ functional outcome was defined as a GOS-E Peds category 1 or 2, indicating no assistance needed for daily activities, behave age appropriately outside the home, able to function at work or in school at his or her previous capacity, resume regular social and leisure activities, and maintenance of relationships with family and friends. A ‘poor’ functional outcome corresponded to categories 3–8. Scoring was given by the researchers based on detailed information that was documented in the medical reports. Functional scores were cross-validated between two independent assessors to minimize errors.

The proportion of missing data values in this study was 0.29% with data missing completely at random (Little’s MCAR test p-value 0.096). Therefore, the missing data were not excluded from the analysis. Multivariate logistic regression was used to examine associations between GOS-E Peds scores and various independent variables in groups with sufficient sample size. In this study, this analysis was conducted for the older adolescent group. Variables with significant p-values (<0.05) from the univariate analysis were then included in the multivariate logistic regression model, in which the forward stepwise likelihood ratio (LR) method was applied. Due to small sample sizes, only univariate analysis was performed for the adolescent group (10–14-year-olds).

As this was a retrospective study, a formal sample size calculation was not performed prior to data collection. However, a post-hoc consideration was conducted to assess the adequacy of the final sample, which is presented in the results.

## Results

The sociodemographic and clinical characteristics of all subjects are presented in Table 1. A total of 542 subjects were included in the study. The group with the highest number of participants was the older adolescents age group. The majority of the participants were male (81.5%), with a male-to-female ratio of 4:1. The mean duration between the RTAs and report review was 20.7 ± 14.9 months. The pre-hospital variables were not included as these data were mostly unavailable in the medical reports. Access to initial acute hospital records, which might have contained this information, was not obtainable. Most of these RTA cases occurred in the central region of Malaysia which includes Kuala Lumpur, the capital city of Malaysia. This region is more densely populated, more urbanised and has higher vehicle density.

**Table 1:** The sociodemographic and clinical characteristics of participants.

Characteristic	Value		
*Sociodemographic*	**Young children (7–9-year-olds)**	**Adolescents (10–14-year-olds)**	**Older adolescents (15–17-year-olds)**
**Total participants (n)**	58	149	335
**Functional outcomes, n (%)**			
Good outcome (GOS-E Peds 1–2)	20 (34.5)	35 (23.5)	94 (28.1)
Poor outcome (GOS-E Peds 3–8)	38 (65.5)	114 (76.5)	241 (71.9)
**Mean age at accident ± SD (years)**	8.0 ± 0.8	12.7 ± 1.4	16.1 ± 0.9
**Mean age at review ± SD (years)**	10.0 ± 2.1	14.6 ± 1.9	17.9 ± 1.5
**Mean duration post-RTAs to review ± SD (months)**	23.96 ± 21.7	21.5 ± 14.7	19.8 ± 13.5
**Gender distribution, n (%)**			
Male	35 (60.3)	114 (76.5)	293 (87.5)
Female	23 (39.7)	35 (23.5)	42 (12.5)
**Male-to-female ratio**	1.5: 1	3.3: 1	7: 1
**Ethnic, n (%)**			
Malay	45 (77.6)	123 (82.5)	272 (81.2)
Chinese	2 (3.4)	12 (8.1)	21 (6.3)
Indian	6 (10.3)	14 (9.4)	31 (12.5)
Others	5 (8.7)	0 (0.0)	0 (0.0)
**Pre-injury studying/working status, n (%)**			
Neither studying nor working	0 (0.0)	2 (1.3)	4 (1.2)
Studying	58 (100)	144 (96.7)	281 (83.9)
Working	0 (0.0)	3 (2.0)	50 (14.9)
**Pre-injury education level, n (%)**			
Primary	58 (100)	62 (41.6)	13 (3.9)
Secondary	0 (0.0)	87 (58.4)	316 (94.9)
Tertiary	0 (0.0)	0 (0.0)	4 (1.2)
Nil	0 (0.0)	0 (0.0)	0 (0.0)
**Mean years of education ± SD (years)**	2.1 ± 1.31	6.5 ± 1.6	9.6 ± 1.36
*Injury details*			
**Place of RTAs** ^a^ **, n (%)**			
Central region	27 (46.5)	67 (45.0)	160 (47.8)
Northern region	3 (5.2)	2 (1.3)	13 (3.9)
East coast region	23 (39.7)	50 (33.6)	105 (31.3)
Southern region	5 (8.6)	30 (20.1)	57 (17.0)
**Mode of RTAs, n (%)**			
Car	14 (24.2)	22 (14.8)	10 (3.0)
Motorcycle	12 (20.7)	90 (60.4)	309 (93.1)
Lorry	2 (3.4)	0 (0.0)	0 (0.0)
Pedestrian	22 (37.9)	20 (13.4)	11 (3.3)
Others	8 (13.8)	17 (11.4)	2 (0.6)
**Severity of TBI, n (%)**			
Mild	17 (32.1)	38 (27.7)	103 (32.1)
Moderate	7 (13.2)	30 (21.9)	82 (25.5)
Severe	29 (54.7)	69 (50.4)	136 (42.4)
**Neurosurgical intervention, n (%)**			
No	41 (70.7)	86 (57.7)	228 (68.1)
Yes	17 (29.3)	63 (42.3)	107 (31.9)
**Presence of concomitant injury**^b^, **n (%)**			
No	7 (12.1)	25 (16.8)	40 (11.9)
Yes	51 (87.9)	124 (83.2)	295 (88.1)
**Intervention for concomitant injury, n (%)**			
No	26 (44.8)	83 (55.7)	138 (41.2)
Yes	32 (55.2)	66 (44.3)	197 (58.8)
**ICU admission, n (%)**			
No	27 (46.6)	83 (55.7)	182 (54.5)
Yes	31 (53.4)	66 (44.3)	152 (45.5)
**Presence of acute medical issues during admission**^c^, **n (%)**			
No	55 (94.8)	132 (89.9)	315 (94.0)
Yes	3 (5.2)	17 (10.1)	20 (6.0)
**Tracheostomy, n (%)**			
No	51 (87.9)	122 (81.9)	269 (80.3)
Yes	7 (12.1)	27 (18.1)	66 (19.7)
**Mean length of ICU Admission ± SD (days)**	9.5 ± 4.9*	7.5 ± 4.8^#^	8.2 ± 4.8^@^
**Mean length of Hospital Stay ± SD (days)**	21.3 ± 31.01	19.7 ± 32.8	16.7 ± 21.9
*Post-TBI complications*			
**Post-traumatic headache, n (%)**			
No	30 (51.7)	72 (48.3)	148 (44.2)
Yes	28 (48.3)	77 (51.7)	187 (55.8)
**Pain in other body parts, n (%)**			
No	56 (96.6)	126 (84.6)	279 (83.3)
Yes	2 (3.4)	23 (15.4)	56 (16.7)
**Post-traumatic seizure, n (%)**			
No	46(79.3)	129 (86.6)	288 (86.0)
Yes	12 (20.7)	20 (13.4)	47 (14.0)
**Behavioural issues and types, n (%)**			
Nil	38 (65.5)	71 (47.6)	160 (47.8)
Angry easily	6 (10.4)	39 (26.2)	73 (21.8)
Withdrawn	9 (15.5)	17 (11.4)	37 (11.0)
Developmentally inappropriate	4 (6.9)	14 (9.4)	48 (14.3)
Mixed	1 (1.7)	8 (5.4)	17 (5.1)
*Medication*			
**Prescribed anti-epileptic medications, n (%)**			
No	35 (60.3)	140 (94.0)	316 (94.3)
Yes	23 (39.7)	9 (6.0)	19 (5.7)

Abbreviations: GOS-E Peds, Glasgow Outcome Scale Extended Pediatric; RTAs, road traffic accidents; TBI, traumatic brain injury; ICU, intensive care unit.

^a^Grouped based on regions in west Malaysia.

^b^Include all other injuries involving neck, facial, chest, abdominal, spine, upper extremities, lower extremities and external injury.

^c^Included, but were not limited to, pneumonia, sepsis, and surgical site infections.

* only 12 out of 31 records were available for analysis.

^**#**^ only 26 out of 66 records were available for analysis.

@only 67 out of 152 records were available for analysis.

Overall, only 27.5% of participants reported good functional outcomes (GOS-E Peds 1 or 2) at review, defined as independent in activities of daily living, behave age appropriately outside the home, able to function at work or in school at his or her previous capacity, resume regular social and leisure activities, and ability to maintain relationships with family and friends. The highest percentage of good outcomes was observed in young children (34.5%), followed by older adolescents (28.0%) and adolescents (23.5%).

In addition to the primary analysis, a post-hoc power analysis was conducted. Based on the observed prevalence of good functional outcome (27.5%) as measured by the GOS-E Peds, and applying a 5% absolute precision with a 95% confidence level, the minimum required sample size was estimated at 307 participants. The actual sample size included in this study was 542, which exceeds this requirement and supports the precision and stability of the estimated outcome proportions.

**Fig 1:**
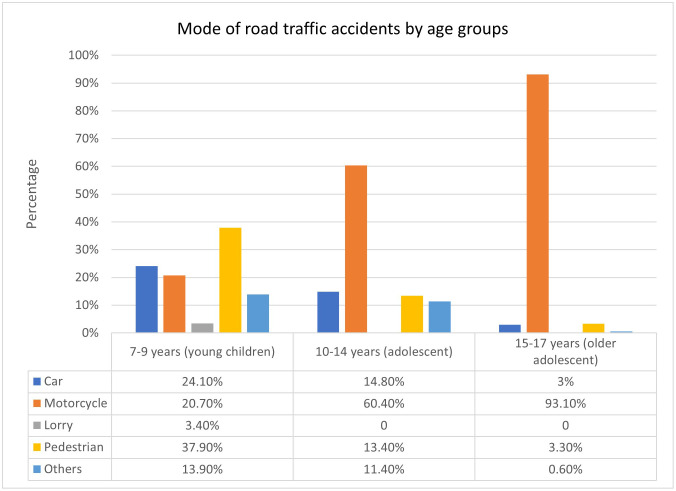
Mode of road traffic accidents by age groups.

Motorcycle-related RTAs were the most common mode of injury in this study, accounting for 76.3% of all RTAs. The majority of the motorcycle-related cases involved older adolescents (75.0%) and adolescents (22.0%). Among young children, pedestrian injuries were the most common cause of TBI (Fig 1). Of the 411 motorcycle-related RTA cases, 298 involved riders and 112 involved pillion passengers. However, documentation regarding helmet use was incomplete in the majority of records. The mean age at the time of accident was 15.7 years, with the youngest motorcycle rider aged 11. The legal minimum age to obtain a motorcycle driving license in Malaysia (Class B and B2) is 16 years. [18] In this study, 36% of motorcycle riders were under the legal age at the time of the accident.

Common complaints reported by participants or caregivers at the point of review were forgetful, headache, and dizziness. These findings are consistent in all three age groups. Other complaints reported were difficulty sleeping, vision problems (blurred, double vision, etc.), speech problems, difficulty walking and impaired concentration.

### Outcomes in young children (7- to 9-year-olds)

This cohort comprised 58 children with a mean age of 7.9 ± 0.8 years, including 35 males (60.3%) and 23 females (39.7%). The majority were of Malay ethnicity (77.6%), followed by Indian (10.3%), Chinese (3.4%), and other ethnicities. On average, the children had completed two years of formal education prior to the accident. The most common type of RTA in this group involved pedestrian injuries (37.9%), followed by car-related accidents (24.2%), motorcycle-related accidents (20.7%), and other causes (Fig 1). Among the pedestrian cases, eight children sustained injuries while walking to or from school, with many incidents occurring while crossing roads or playing near their homes.

In this cohort, 54.7% sustained severe TBI, 32.1% had mild TBI, and 13.2% had moderate TBI. Neurosurgical interventions were performed in 29.3% of cases. Of the 58 subjects, 51 (87.9%) had concomitant injuries; 32 (62.7%) of them underwent intervention. Twenty-six subjects (51.0%) had concomitant injuries affecting more than two body regions, and intervention was required in 22 (84.6%) subjects. Regarding post-TBI complications, 48% reported post-traumatic headaches, 3.4% experienced significant pain in other body regions, and 21% developed post-traumatic seizures, with 8.6% on anti-epileptic medications. Behavioural issues were noted in 34% participants, including irritability or anger (n = 6), social withdrawal (n = 6), age-inappropriate behaviour (n = 4), and multiple behavioural issues (n = 1).

Prior to their TBI, all children were already enrolled in schools, including one child in kindergarten. After the injury, 88% returned to school, although 67% of them experienced academic difficulties such as declining performance. Four students were subsequently placed in special education classes or transferred to special schools. Conversely, 22% did not return to school. Among them, three remained in a disorder of consciousness, three were unable to return due to physical disabilities, and one child did not return for an unspecified reason. Due to the small sample size, no further analysis was performed.

### Outcomes in adolescent (10- to 14-year-olds)

This group consisted of 149 adolescents with a mean age of 12.7 ± 1.41 years and the majority were male (76.5%). On average, they had 6.5 ± 1.6 years of formal education. Majority were of Malay ethnicity (82.6%), 9.4% were Chinese and 8.4% were Indian. Most of the accidents took place in the central region of Malaysia (45.0%). The most common type of RTAs in this group involved motorcycles (60.4%), followed by car-related accidents (14.8%), pedestrian (13.4%) and others (Fig 1).

Among the participants in this group, 50.4% sustained severe TBI, followed by 27.7% with mild TBI and 21.9% with moderate TBI. Neurosurgical interventions were performed in 42.3% of the cases. Of the 149 subjects, 142 (83.2%) sustained concomitant injuries, of whom 66 (46.5%) underwent related interventions. Among those, 59 (41.5%) had concomitant injuries affecting more than two body regions, and intervention was required in 42 (71.2%) subjects. Additionally, 10% experienced acute medical complications during the initial admission post-injury, and 18% required tracheostomy. Post-TBI complications included post-traumatic headache (51.7%), significant pain in other body regions (15.4%), and post-traumatic seizures (13.4%) with 6% on anti-epileptic medications. Behavioural issues were observed in 52.3% of participants.

Before sustaining TBI, 144 were studying (41.4% in primary school while 58.4% in secondary school), three were employed, and two were neither studying nor working. At review, 118 adolescents (79.2%) returned to school. However, 65% reported challenges, primarily involving deteriorated academic performance, while one participant skipped school due to pain, and two eventually transferred to special schools. Of the three participants employed pre-TBI, all returned to their jobs. Additionally, seven participants who were studying pre-TBI transitioned to employment by the time of review, mostly in odd jobs or labour work, with only one working as a clerk.

Univariate analysis of sociodemographic and clinical characteristics and their associations with functional outcomes in this subgroup are presented in Table 2. However, a multivariate analysis could not be performed due to the limited sample size. Independent variables significantly associated with functional outcomes (p < 0.05) included pre-injury studying or working status, TBI severity, neurosurgical intervention, and the presence of acute medical complications during the initial admission.

**Table 2:** Univariate analysis of sociodemographic and clinical characteristic and their association with functional outcomes for adolescent group (10 to 14-year-olds).

Independent variable	n (%)	Good outcome^a^ n (%)	Poor outcome^b^ n (%)	p-value
**Duration of TBI**				
2 years and below	110 (73.8)	26 (23.6)	84 (76.4)	0.944
> 2 years	39 (26.2)	9 (23.1)	30 (76.9)	
**Gender**				
Male	114 (76.5)	26 (22.8)	88 (77.2)	0.723
Female	35 (23.5)	9 (25.7)	26 (74.3)	
**Ethnicity**				
Malay	123 (82.6)	27 (22.0)	96 (78.0)	0.603
Chinese	12 (8.1)	4 (33.3)	8 (66.7)	
Indian	14 (9.4)	4 (28.6)	10 (71.4)	
Others	0	0 (0.0)	0 (0.0)	
**Pre-injury studying/working status**				
Neither studying nor working	2 (1.3)	2 (100)	0 (0.0)	**0.007**
Studying	144 (96.6)	31 (21.5)	113 (78.5)	
Working	3 (2.0)	2 (66.7)	1 (33.3)	
**Education Level**				
Primary	62 (41.6)	19 (30.6)	43 (69.4)	0.082
Secondary	87 (58.4)	16 (18.4)	71 (81.6)	
**Place of accident by region**				
Central region	67 (45.0)	18 (26.9)	49 (73.1)	0.728
Northern region	2 (1.3)	0 (0.0)	2 (100.0)	
East coast region	50 (33.6)	11 (22.0)	39 (78.0)	
Southern region	30 (20.1)	6 (20.0)	24 (80.0)	
**Mode of RTAs**				
Car	22 (14.8)	9 (40.9)	13 (59.1)	0.053
Motorcycle	90 (60.4)	18 (20.0)	72 (80.0)	
Pedestrian	20 (13.4)	2 (10.0)	18 (90.0)	
Others	17 (11.4)	6 (35.3)	11 (64.7)	
**Severity of TBI**				
Mild	38 (27.7)	14 (36.8)	24 (63.2)	**0.001**
Moderate	30 (21.9)	11 (36.7)	19 (63.3)	
Severe	69 (50.4)	7 (10.1)	62 (89.9)	
**Neurosurgical Intervention**				
No	86 (57.7)	27 (31.4)	59 (68.6)	**0.008**
Yes	63 (42.3)	8 (12.7)	55 (87.3)	
**Presence of concomitant injury**				
No	25 (16.8)	8 (32.0)	17 (68.0)	0.271
Yes	124 (83.2)	27 (21.8)	97 (78.2)	
**Intervention for concomitant injury**				
No	83 (55.7)	21 (25.3)	62 (74.7)	0.559
Yes	66 (44.3)	14 (21.2)	52 (78.8)	
**ICU admission**				
No	83 (55.7)	24 (28.9)	59 (71.1)	0.08
Yes	66 (44.3)	11 (16.7)	55 (83.3)	
**Presence of acute medical issues during admission**				
No	132 (89.9)	35 (26.5)	97 (73.5)	**0.015**
Yes	17 (10.1)	0 (0.0)	17 (100.0)	
**Tracheostomy**				
No	122 (81.9)	32 (26.2)	90 (73.8)	0.094
Yes	27 (18.1)	3 (11.1)	24 (88.9)	
**Post-traumatic headache**				
No	72 (48.3)	20 (27.8)	52 (72.2)	0.233
Yes	77 (51.7)	15 (19.5)	62 (80.5)	
**Pain at other body parts**				
No	126 (84.6)	29 (23.0)	97 (77.0)	0.749
Yes	23 (15.4)	6 (26.1)	17 (73.9)	
**Post-traumatic seizure**				
No	129 (86.6)	33 (25.6)	96 (74.4)	0.126
Yes	20 (13.4)	2 (10.0)	18 (90.0)	
**Types of behaviour issue**				
Nil	71 (47.7)	19 (26.8)	52 (73.2)	0.457
Angry easily	39 (26.2)	10 (25.6)	29 (74.4)	
Withdrawn	17 (11.4)	4 (23.5)	13 (76.5)	
Developmentally inappropriate	14 (9.4)	2 (14.3)	12 (85.7)	
Mixed	8 (5.4)	0 (0.0)	8 (100.0)	
**Prescribed anti-epileptic medications**				
No	140 (94.0)	35 (25.0)	105 (75.0)	0.086
Yes	9 (6.0)	0 (0.0)	9 (100.0)	

Abbreviations: RTAs, road traffic accidents; TBI, traumatic brain injury; ICU, intensive care unit.

^a^GOS-E Peds 1–2.

^b^GOS-E Peds 3–8.

### Outcomes in older adolescent (15- to 17-year-olds)

This group comprised 335 older adolescents aged 15–17 years, with a mean age of 16.1 ± 0.86 years. 87.5% were male (Table 3). On average, they had completed 9.6 ± 1.36 years of formal education. Majority were of Malay ethnicity (81.2%), 12.5% were Indian and the remaining were Chinese. Most of these accidents happened in the central region (47.8%), followed by the northern and east coast region (32.5%). The most common type of RTAs in this group involved motorcycles (93.0%), followed by pedestrian (3.3%).

**Table 3:** Univariate analysis of sociodemographic and clinical characteristic and their association with functional outcomes for older adolescent group (15 to 17-year-olds).

Independent variable	n (%)	Good outcome^a^ n (%)	Poor outcome^b^ n (%)	p-value^c^
**Duration of TBI**				
2 years and below	251 (74.9)	65 (25.9)	186 (74.1)	0.128
> 2 years	84 (25.1)	29 (34.5)	55 (65.5)	
**Gender**				
Male	293 (87.5)	81 (27.6)	212 (72.4)	0.655
Female	42 (12.5)	13 (31.0)	29 (69.0)	
**Ethnicity**				
Malay	272 (81.2)	76 (27.9)	196 (72.1)	0.834
Chinese	21 (6.3)	7 (33.3)	14 (66.7)	
Indian	31 (12.5)	11 (26.2)	31 (73.8)	
Others	0 (0.0)	0 (0.0)	0 (0.0)	
**Pre-injury studying/working status**				
Neither studying nor working	4 (1.2)	1 (25.0)	3 (75.0)	0.596
Studying	281 (83.9)	76 (27.0)	205 (73.0)	
Working	50 (14.9)	17 (34.0)	33 (66.0)	
**Education Level**				
Primary	13 (3.9)	5 (38.5)	8 (61.5)	0.076
Secondary	316 (94.9)	86 (27.2)	230 (72.8)	
Tertiary	4 (1.2)	3 (75.0)	1 (25.0)	
**Place of accident by region**				
Central region	160 (47.8)	51 (31.9)	109 (68.1)	**0.033**
Northern and east coast region	118 (35.2)	23 (19.5)	95 (80.5)	
Southern region	57 (17.0)	20 (35.1)	37 (64.9)	
**Mode of MVA**				
Car	10 (3.0)	5 (50.0)	5 (50.0)	0.184
Motorcycle	309 (93.1)	86 (27.8)	223 (72.2)	
Pedestrian	11 (3.3)	1 (9.1)	10 (90.9)	
Others	2 (0.6)	1 (50.0)	1 (50.0)	
**Severity of TBI**				
Mild	103 (32.1)	43 (41.7)	60 (58.3)	**<0.001**
Moderate	82 (25.5)	26 (31.7)	56 (68.3)	
Severe	136 (42.4)	20 (14.7)	116 (85.3)	
**Neurosurgical Intervention**				
No	228 (68.1)	74 (32.5)	154 (67.5)	**0.009**
Yes	107 (31.9)	20 (18.7)	87 (81.3)	
**Presence of concomitant injury**				
No	40 (11.9)	9 (22.5)	31 (77.5)	0.404
Yes	295 (88.1)	85 (28.8)	210 (71.2)	
**Intervention for concomitant injury**				
No	138 (41.2)	43 (31.2)	95 (68.8)	0.291
Yes	197 (58.8)	51 (25.9)	146 (74.1)	
**ICU admission**				
No	182 (54.5)	67 (36.8)	115 (63.2)	**<0.001**
Yes	152 (45.5)	27 (17.8)	125 (82.2)	
**Presence of acute medical issues during admission**				
No	315 (94.0)	93 (29.5)	222 (70.5)	**0.018**
Yes	20 (6.0)	1 (5.0)	19 (95.0)	
**Tracheostomy**				
No	269 (80.3)	90 (33.5)	179 (66.5)	**<0.001**
Yes	66 (19.7)	4 (6.1)	62 (93.9)	
**Post-traumatic headache**				
No	148 (44.2)	47 (31.8)	101 (68.2)	0.18
Yes	187 (55.8)	47 (25.1)	140 (74.9)	
**Pain at other body parts**				
No	279 (83.3)	77 (27.6)	202 (72.4)	0.675
Yes	56 (16.7)	17 (30.4)	39 (69.6)	
**Post-traumatic seizure**				
No	288 (86.0)	85 (29.5)	203 (70.5)	0.143
Yes	47 (14.0)	9 (19.1)	38 (80.9)	
**Behavioural issues and types**				
Nil	160 (47.8)	60 (37.5)	100 (62.5)	**0.004**
Angry easily	73 (21.8)	17 (23.3)	56 (76.7)	
Withdrawn	37 (11.0)	7 (18.9)	30 (81.8)	
Developmentally inappropriate	48 (14.3)	6 (12.5)	42 (87.5)	
Mixed	17 (5.1)	4 (23.5)	13 (76.5)	
**Prescribed anti-epileptic medications**				
No	316 (94.3)	94 (29.7)	222 (70.3)	**0.005**
Yes	19 (5.7)	0 (0.0)	19 (100.0)	

Abbreviations: RTAs, road traffic accidents; TBI, traumatic brain injury; ICU, intensive care unit.

^a^GOS-E Peds 1–2.

^b^GOS-E Peds 3–8.

^c^p-values of <0.05 were included in the multivariate logistic regression.

Among the participants, 42.4% sustained severe TBI, 32.1% had mild TBI, and 25.5% experienced moderate TBI. In this cohort of 335 subjects, concomitant injuries were observed in 295 cases (88.1%), with 197 (66.8%) requiring intervention. Concomitant injuries affecting more than two body regions were documented in 193 subjects (65.1%), of whom 164 (85.0%) underwent intervention. Less than one-third (19.7%) required tracheostomy. Reported post-TBI complications included post-traumatic headache (55.8%), significant pain in other body regions (16.7%), and post-traumatic seizures (14.0%) with 5.7% prescribed anti-epileptic medications. Behavioural disturbances were observed in 52.2% of participants.

Before sustaining TBI, 281 subjects were studying, 50 were employed, and four were neither studying nor working. At review, 211 subjects (63.0%) returned to school. However, 59% experienced challenges after returning, such as deteriorated academic performance and difficulty coping. Of these, 19 eventually dropped out, failed their national examinations, or transferred to special schools. Additionally, 20 participants returned to work post-TBI, while another 27 who were previously studying became gainfully employed, marking a notable shift in employment status.

Table 3 shows univariate analysis of sociodemographic and clinical characteristics and their association with functional outcomes. Variables that were significant in the univariable logistic regression (P < 0.05) were included for variable selection in multivariate logistic regression. There are eight variables that were significantly correlated with functional outcome in the univariate analysis as shown in Table 3: place of accident by region, severity of TBI, neurosurgical intervention, ICU admission, presence of acute medical issues during admission, tracheostomy, anti-epileptic drug prescription and behaviour issue. The forward stepwise likelihood ratio (LR) method was then applied, which initially retained five variables. However, as the odds ratio (OR) for one variable was not estimable, this variable was removed, and the selection process was repeated. The final model retained three variables, as presented in Table 4.

**Table 4:** Multivariate logistic regression of the significant independent variables with good functional outcomes based on GOS-E Peds.

Independent variables	OR	95% CI	p value
***Severity of TBI***			**0.021** ^a^
Mild TBI	2.5	1.29-4.83	**0.007**
Moderate TBI	1.77	0.87-3.6	0.114
Severe TBI			Ref.
***Tracheostomy (No)***	7.02	2.06-23.93	**0.002**
***Types of behaviour issue***			**0.005** ^a^
Nil	2.41	0.72-8.03	0.152
Angry easily	1.12	0.30-4.16	0.861
Withdrawn	0.93	0.22-3.88	0.915
Developmentally inappropriate	0.54	0.12-2.42	0.422
Mixed			Ref.

Abbreviations: OR, odds ratio; CI, confidence interval; TBI, traumatic brain injury.

^a^P-value at variable level based on forward logistic regression (LR) test.

Table 4 found that mild TBI patients are 2.5 times more likely to have a good functional outcome compared to patient with severe TBI (OR = 2.5; 95% CI 1.29–4.83) and patient who does not require tracheostomy after TBI had 7 times the odds of having a good functional outcome than those who required a tracheostomy (OR = 7.02; 95% CI: 2.06–23.93). Absence of behaviour issue is associated with better functional outcome; however, this variable is not statistically significant (OR = 2.4, 95% CI: 0.72–8.03). The logistic regression model was statistically significant with p < 0.05 with a nonsignificant Hosmer and Lemeshow test. The model explained 22% (Nagelkerke R2) of the variance in functional outcome and the accuracy of this model is 75.3%

## Discussion

In Southeast Asia, TBI among children is predominantly caused by RTAs, with motorcycles being the most common vehicle involved. [19,20] This profile contrasts with that of developed countries, where the aetiology of paediatric TBI is more varied. In younger children, falls and abusive head trauma are frequently reported, while adolescents more commonly sustain TBIs from motorcycle, bicycle, and sports-related injuries. [21] The proportion of paediatric TBIs linked to RTAs in Malaysia is among the highest in the region, reaching up to 95.4%. [2,22] Despite this burden, regional data on functional outcomes following paediatric TBI remain limited.

Motorcycle-related RTAs emerged as the most prevalent mode of injury in this study, with a significant proportion (75%) involving older adolescents. The widespread use of motorcycles among adolescents is often accompanied by inadequate helmet use and limited road safety awareness. In this study, the prevalence of helmet use could not be determined, as this information was not documented in the available reports. Notably, nearly one-third of motorcycle riders were under the legal age for obtaining a motorcycle license at the time of the accident.

Underage motorcycle riding is a prevalent regional public health concern, not one unique to Malaysia. [23,24] For instance, reports from Vietnam identify high school-aged children as the most vulnerable group for traffic accidents, with a high proportion being underage motorcycle riders. [25] This high incidence of RTA-related TBI among children has prompted national responses across the region. Thailand, for example, introduced the Safe Road Projects initiative in 2025 to reduce youth traffic injuries. Similarly, Malaysia integrated compulsory Road Safety Education into its primary school national curriculum by 2010.

However, addressing the issue of illegal underage motorcycle riding remains challenging, as many adolescents receive parental consent to ride. A study from Vietnam highlighted that this behaviour is often socially accepted or considered the norm in certain communities, contributing to its continued prevalence. [24] This underscores the need for injury prevention strategies in this age group to extend beyond legal enforcement. Effective approaches must incorporate not only school-based road safety education but also community involvement, as well as increased parental accountability.

Consistent with previous Malaysian studies on paediatric TBIs, we found no statistically significant difference in outcomes across age groups. [12,14] While younger children (7–9 years) showed the highest proportion of good recovery in this study, this difference was not statistically significant. This finding appears to contrast with previous literature suggesting that younger children under age six generally experience poorer functional outcomes due to ongoing brain maturation. [6–8] Our results may indicate that school-aged children, particularly those older than six, are more cognitively and behaviourally equipped to engage with rehabilitation and benefit from structured educational environments that facilitate early support and reintegration. Further research is needed to confirm this. Our findings are also influenced by the uneven sample distribution across the age categories and variability in follow-up timing, limiting the statistical power to detect subtle differences. Regional studies from Thailand and Vietnam similarly report mixed findings regarding age as a prognostic factor, indicating a need for a standardised outcome tracking system. [22,26]

Greater TBI severity has consistently been associated with poorer outcomes in both regional and global studies, a finding supported by our results. Among older adolescents in our cohort, those with mild TBI were approximately 2.5 times more likely to achieve a good functional outcome than those with severe injuries, indicating a significant association between mild injury and favourable recovery. This aligns with regional data, such as a study of 218 paediatric TBI patients in the Philippines, which reported that only 35% of those with severe TBI had a good outcome at hospital discharge. [11]

In contrast, neither sociodemographic factors such as ethnicity and gender; nor clinical factors such as mechanism of injury, were significantly associated with outcomes in this paediatric cohort. This finding diverges from our previous study of the adult cohort within the same study population, which identified gender, ethnicity, marital status, and education level as factors significantly affecting functional outcome. [15] It is important to note, however, that the adult cohort was considerably larger (n = 1939), which may account for the differences in the variables reaching statistical significance.

An important indicator of functional recovery in school-aged children is the rate of return to education, which we found to be notably high across all three groups. However, a substantial proportion continued to face academic challenges that adversely affected their studies. Almost two-thirds of the children from all three groups were not able to maintain their academic performance pre-injury. Frequently reported issues such as forgetfulness, headaches, and dizziness may have contributed to these difficulties. Additionally, half of the participants reported behavioural problems, which could further hinder their reintegration into the educational environment and affect their long-term academic performance. These findings highlight the importance of routine post-discharge screening as recommended by the International Paediatric Brain Injury Society. [27]

To effectively address the physical, cognitive, emotional and behavioural challenges faced by children following brain injury, a coordinated multidisciplinary response is essential. At the time of hospital discharge, many patients have yet to achieve their optimal level of recovery, making continued care and rehabilitation essential. Early identification of a patient’s needs at discharge allows for targeted planning and timely interventions that support long-term functional outcomes. However, in Malaysia, post-discharge rehabilitation services for the paediatric population remain limited, especially when compared to services available for adults. This is exacerbated by the limited dedicated paediatric TBI inpatient rehabilitation wards throughout the country. This service gap can lead to fragmented care, delayed support, and suboptimal reintegration into school and daily life. [28]

Cognitive deficits, including impairments in memory and learning, alongside behavioural issues, have been well documented in previous studies and are known to contribute to declining school performance following brain injury. [29–32] Although studies from other countries within the region have described the clinical profiles of children with TBI, data specifically examining school reintegration outcomes still remain scarce. [11,25,33] In our study, we observed a significant decline in school performance whereby some of the subjects had to be transferred to special education classes or schools for children with learning disabilities. Among older adolescents, a small proportion discontinued school entirely.

Educational support for children with learning difficulties in Malaysia includes government-funded and private centres aimed at early education assistance. [34,35] Special education programmes are available nationwide which include special education schools, special education integration programme and inclusive education programmes. [35] In addition, the Department of Social Welfare under the Ministry of Women, Family and Community Development offers pre-vocational training and rehabilitation programmes. [35] Despite these provisions, there are gaps in structured reintegration plans for children returning to school post-TBI. These areas warrant attention to ensure comprehensive and multidisciplinary support.

### Limitations of the study

This study has several limitations. Its retrospective design limits the ability to establish causality or capture detailed psychosocial outcomes. Variability in clinical documentation restricted the availability of key details, including rehabilitation interventions and prehospital factors such as helmet use, rider versus pillion status, specifics of the injury mechanism, and emergency response. These gaps limited our ability to fully evaluate modifiable predictors of outcome.

While missing data were minimal and handled appropriately, a prospective design with standardised follow-up and comprehensive data collection would offer greater analytic power. We also acknowledge the methodological compromise of using the GOS-E Peds beyond its validated age range of 16 years to maintain consistency across all subjects. Finally, our statistical approach was largely univariate. The lack of association between time since injury and outcome may also reflect bias introduced by variation in timing of the post-injury assessments.

## Conclusion

In conclusion, this study highlights that motorcycle-related TBIs are common among school-aged children in Malaysia, although there were no significant changes in outcome among the different age groups. A substantial proportion of children experience poor functional outcomes following RTA-related TBI, particularly in maintaining pre-injury academic performance despite returning to school. These findings underscore the need for integrated clinical, educational, and policy frameworks to support children recovering from TBI. In regions like Malaysia, where motorcycle-related injuries are common and paediatric rehabilitation infrastructure is limited, context-specific data such as this can inform scalable, multidisciplinary interventions and help bridge the gap between hospital discharge and long-term reintegration into daily life and education.

## Supporting information

S1 File Paeds TBI Dataset_anonymous. (XLSX)
