# Supplementary material for: Outcomes of school-aged children following traumatic brain injury from road traffic accidents in Malaysia: A retrospective study
Source: PLoS One. 2026 Jun 16;21(6):e0351136. doi: 10.1371/journal.pone.0351136 (PMC13271509; doi:10.1371/journal.pone.0351136)
Supplement: Attachment — Submitted filename: Response to Reviewers.docx [file pone.0351136.s002.docx]

**Response to Reviewers (PLoS One)**

**Outcomes of school-aged children following traumatic brain injury from road traffic accidents in Malaysia: a retrospective study**

**Dear Editor**

|  | Comments | Action |
| --- | --- | --- |
| 1. | The title is very broad, a more specific title, stressing on the setting, is encouraged | “Malaysia” has been added to the title to make it more specific:  Outcomes of school-aged children following traumatic brain injury from road traffic accidents in Malaysia: a retrospective study |
| 2. | The details concerning how the sample size calculation was done | Thank you for your comment. As this was a retrospective observational study, a formal sample size calculation was not performed prior to data collection. However, to address concerns regarding adequacy of sample size, we conducted a post-hoc power consideration based on the observed prevalence of good functional outcome.  In our study, 27.5% of school-aged children demonstrated good functional outcomes following TBI, as measured by the GOS-E Peds. Using this observed prevalence, we estimated that a sample size of 307 would be required to estimate the outcome proportion with a 5% absolute precision at a 95% confidence level. Our achieved sample size of 542 participants exceeds this requirement, supporting the precision and reliability of the outcome estimate.  We have now included this justification in the manuscript under the Methods and Results section. Page 8 and 9 |
| 3. | the steps and variables of the multivariate statistical analysis | We have further clarified the steps for the multivariate analysis in the method as well as result section. Page 8 and 13  Variables that were significant in the univariable logistic regression (P < 0.05), as shown in Table 3, were included for variable selection. We applied the forward stepwise likelihood ratio (LR) method, which initially retained five variables. However, as the odds ratio (OR) for one variable was not estimable, this variable was removed, and the selection process was repeated. The final model retained three variables, which we presented in table 4. |
| 4. | In the results section, why the prehospital variables haven't been mentioned | The pre-hospital variables were not included as these data were not available in the medical reports. Access to initial acute hospital records, which might have contained this information, was not obtainable.  We have briefly mentioned this in the result section (Page 8,9) and added this in the limitation |
| 5. | Discussion should be more focus on the regional features of the study population and the potentially impacts on the local policies. The discussion section lacks the comparison with local and regional findings | We have re-written the manuscript to add more comparisons with available data in the region |

Reviewer #1

|  | Comments | Action |
| --- | --- | --- |
| 1. | Specifying the context in the title would be good. | “Malaysia” has been added to the title to make it more specific:  Outcomes of school-aged children following traumatic brain injury from road traffic accidents in Malaysia: a retrospective study |
| 2. | I feel the conclusion that provision of "age-targeted road safety education" is not a direct outcome of this study but rather the study findings support the continuance of existing programs that do this already. | We have edited the conclusion both in the abstract and in the text (under conclusion) to make it more align with the outcome of this study which include emphasizing the need for integrated clinical, educational, and policy frameworks to support children recovering from TBI |
| 3. | Does the proportion of Motorcycles as the vehicle involved in the RTA resulting in TBI reflect the proportion of motorcycles on the roads? | Yes, the proportion of Motorcycles as the vehicle involved in the RTA resulting in TBI reflect the proportion of motorcycles on the roads in Malaysia. Based on road fatalities by mode (2010 – 2019), highest road fatalities (60 percent) are coming from motorcyclists.  <https://www.mot.gov.my/en/media/annual-report/yearly-statistic>  According to Malaysia transportation statistic 2024 by Ministry of Transport Malaysia, motorcycle amount to 54% of registered vehicle in 2024.  <https://www.mot.gov.my/en/land/safety/malaysia-road-fatalities-index> |
| 4. | Why pre-hospital observations of accident scene, vital signs, interventions, and then more in-hospital diagnosis and procedures were not used as part of the study. As only tracheostomy appeared as a significant factor, it may have been worthwhile considering more granular representations of other variables. For instance ICU Length of Stay. Inclusion of these additional attributes may shed more light on factors contributing to both TBI level and functional recovery | Thank you for this insightful comment. We fully agree that pre-hospital observations and more granular in-hospital variables would have added valuable context to our analysis.  However, as this was a retrospective study based on medical reports, pre-hospital data were often unavailable or inconsistently documented. Many patients were reviewed months to years after their initial injury, and care was often initiated at hospitals across various states in Malaysia. As such, the medical reports were primarily compiled for follow-up purposes, focused primarily on the patient’s current functional status, and relying on brief summaries from referring centres. The absence of a standardised reporting format for pre-hospital variables in our setting resulted in significant variability and missing data across records.  While we recognise the potential value of including ICU length of stay, this information was only available in fewer than half of the ICU cases (n < 125), limiting its utility for meaningful analysis.  We acknowledge this as a limitation of the dataset and have now clarified it in the manuscript. Nonetheless, we believe our findings offer an important starting point, particularly as this is, to our knowledge, the first study in Southeast Asia to extensively examine both functional and school reintegration outcomes in children with TBI.  We hope this work provides a foundation for future research that can incorporate more comprehensive pre-hospital and in-hospital variables through prospective data collection.  We have added in the limitation section and mentioned to incorporate this in future studies |
| 5. | Given the socio-demographic, RTA, and TBI related factors, could a prediction of functional outcome be made, and hence back-to-school or return-to-work support program be recommended based on the anticipated functional outcome. | Thank you for the suggestion. We agree that predictive modelling could be useful for planning post-TBI support. However, given the retrospective nature of this study and variability in available data, our analysis was limited to exploratory associations across three age groups to identify potential factors associated with functional outcomes post-TBI.  Notably, only the oldest age group (15–17 years) showed significant differences in univariate analysis for us to proceed with multivariate analysis. These findings suggest that age-specific patterns may exist, but given the cross-sectional nature of the data and variability in record completeness, we did not pursue predictive modelling at this stage.  Developing a predictive model would require more standardised, prospective data, which we hope future studies can build upon. |
| 6. | I feel that an additional line of investigation may have been pairwise analysis variables (e.g. correlation analysis). For instance, is neurosurgical intervention related to TBI severity? | Thank you for the suggestion.  While correlations analysis between neurosurgical intervention and TBI severity could be of interest, they were not part of our initial study objectives.  In our cohort, neurosurgical intervention was typically performed for patients with severe TBI as most of them are craniectomy and craniotomy with evacuation of clot, and it was shown to have an association with outcome in our initial univariate analysis. However, this association did not remain significant in the multivariate model, likely reflecting the underlying confounding effect of injury severity. We have focused our analysis on variables that independently contribute to outcome, in line with our exploratory aim.  We hope this explanation is satisfactory  However, we found that it is a variable that was associated with the outcomes in univariate analysis and we have added that variable into the final multivariate analaysis. |
| 7. | I also feel that inclusion of more RTA factors may be useful. For instance, in the case of motorcycle accident, was the injured person rider or pillion passenger, was the injured person wearing a helmet, what was the other vehicle involved in the collision.  In our work with RTAs we have found other factors such as vehicle rollover, ejection from the vehicle, fractures, injury to multiple body regions, etc. being predictive of injury severity. Pre-hospital factors are also relevant to injury severity, recovery and subsequent functional outcomes. Tracheostomy pre-hospital is one such indicator. Others include blood transfusion and CPR. | We appreciate the reviewer’s insightful suggestions and fully agree that these RTA-related and pre-hospital factors can influence injury severity and functional outcomes. However, due to the retrospective nature of the study and the variability in medical record completeness, many of these variables were either unavailable or inconsistently documented.  For example, among the motorcycle-related RTAs, we were able to identify 298 riders and 112 pillion passengers. However, more than 50% of the records lacked documentation on helmet use, limiting our ability to analyse its association with outcomes.  Detailed information regarding the circumstances of the accident (e.g. vehicle type, rollover, ejection) and pre-hospital events (e.g. CPR, blood transfusion, tracheostomy before hospital arrival) was largely missing from the records and could not be reliably included in the analysis.  We have added this in the limitation.  That said, we have incorporated additional narrative reporting in the manuscript on the subset of children with concomitant injuries involving two or more body regions, along with the proportion who received interventions for these injuries. |

Reviewer #2

|  | Comments | Action |
| --- | --- | --- |
| 1. | The data availability statement is no complete, as it states that “all relevant data are within the manuscript” while the manuscript only includes aggregated data. Further clarification might be needed on that regard. | Thank you for the comment. We have revised the data availability statement for clarity.  It now reads: “The data that support the findings of this study are only available from the corresponding author upon reasonable request, with all identifying information anonymised to maintain confidentiality. This is partly due to the ethical concerns surrounding sharing medical records, especially those of minors” |
| 2. | There is no information on the sample size calculation, which doesn’t allow the reader to appreciate whether there is enough power for the analyses performed, especially the regression analysis | Thank you for your comment. As this was a retrospective observational study, a formal sample size calculation was not performed prior to data collection. However, to address concerns regarding adequacy of sample size, we conducted a post-hoc power consideration based on the observed prevalence of good functional outcome.  In our study, 27.5% of school-aged children demonstrated good functional outcomes following TBI, as measured by the GOS-E Peds. Using this observed prevalence, we estimated that a sample size of 307 would be required to estimate the outcome proportion with a 5% absolute precision at a 95% confidence level. Our achieved sample size of 542 participants exceeds this requirement, supporting the precision and reliability of the outcome estimate.  We have now included this justification in the manuscript under the Methods and Results section. **Page 8 and 9** |
| 3. | The reason to run the analyses per subgroup instead of including age as a covariate is not clear, especially given the limited sample size in two of the groups. | Thank you for your comment. We chose to conduct subgroup analyses by age group rather than including age as a covariate in a single model, as our aim was to explore whether different age ranges presented with distinct clinical profiles, injury characteristics, and functional outcomes.  Children aged 7 to 17 years span key developmental stages from early school-age to late adolescence, which may influence both injury mechanisms and recovery trajectories. Grouping the cohort into three clinically meaningful age bands (7–9, 10–14, and 15–17 years) allowed us to observe and describe these differences more clearly.  While we acknowledge the limitations of smaller sample sizes in two of the groups, this exploratory approach was intended to generate insights that could inform more targeted research and interventions in future prospective studies.  We acknowledge that the rationale for age-based subgroup analyses was only briefly mentioned in the abstract and methodology. We have now added a more detailed explanation in the methodology to clarify the reasoning behind this approach (**page 7**) |
| 4. | In the regression model run with older adolescents, the variables inclusion process is not clear/does not match what is explained in the methods section.  Indeed, among the 8 factors significantly associated with the outcome in the univariate analysis (table 3), 5 did not seem to be included in the multivariate analysis (Table 4). | We have further clarified the steps for the multivariate analysis in the method (**page 8**) as well as result section (**page 13 and 14**).  Variables that were significant in the univariable logistic regression (P < 0.05), as shown in Table 3, were included for variable selection. We applied the forward stepwise likelihood ratio (LR) method, which initially retained five variables. However, as the odds ratio (OR) for one variable was not estimable, this variable was removed, and the selection process was repeated. The final model retained three variables, which we presented in table 4. |
| 5. | Specifying the context in the title would be good | “Malaysia” has been added to the title to make it more specific:  Outcomes of school-aged children following traumatic brain injury from road traffic accidents in Malaysia: a retrospective study |
| 6. | In the introduction, reference for global data is relatively old and could benefit some updates. | After extensive search, the global epidemiology data specifically for pediatric TBI in LMIC were mostly in the 2000. The one we cited (Dewan MC, 2016) is the latest available global data for LMICs. The incidence of TBI in LMIC is very likely underreported with an even greater gap in Southeast Asia. We decided to maintain that reference but adding a more recent data from WHO in 2023, which looks at global overall pattern of road safety and injuries (Global status report on road safety, 2023) |
| 7. | Moreover, references are sometimes placed at the end, sometimes in the middle of sentences (e.g. line 71), which would be good to harmonize. | We have harmonized the references to be at the end. |
| 8. | The use of routine data is an asset, however it carries also some risks, including selection bias. It would be good to discuss the implications of such data source in the discussion. | We have included his detail in the limitation (**page 18**) |
| 9. | The definition and source of information for the variable on “complication” needs some clarification and consistency between methods and results. | We acknowledge that the manuscript was slightly confusing between acute medical issues during admission and post-TBI complications. Some changes have been made for clearer explanations (**page 6**)  As a clarification:  a) The acute medical issues during admission included, but were not limited to, pneumonia, sepsis, and surgical site infections.  b) Post-TBI complications included post-traumatic headaches, pain, seizures and behavioural problems. |
| 10. | The follow-up period was highly heterogenous (20M +/- 15M). How was this tackled in the analysis? Any sensitivity analyses done to check whether time to injury (at report) was associated with level of recovery? Which implications might it have on findings? | Thank you for raising this important point. We acknowledge the considerable heterogeneity in the time between injury and clinical review. While we did not conduct a formal sensitivity analysis, we explored the relationship between time since injury (at report) and functional outcomes within each age cohort and found no statistically significant association.  This suggests that, in our dataset, the timing of the report review did not meaningfully influence the measured outcomes. Nonetheless, we recognise that this variability may introduce some bias, and we have now noted this in the discussion (**page 16)** |
| 11 | Given the research questions, having the table 1 presented by age groups, including testing differences in terms of sociodemographic and injury characteristics would help the reader to capture those differences and read the results accordingly. | We have made the changes in Table 1 |
| 12 | In Discussion, the findings are not always compared to existing literature (especially in first third of discussion), which weakens interpretation (e.g non significance of given factors, return to school and experienced difficulties,..) | We have re-written the discussion to compare with existing literature, especially with countries around SEA region – as suggested by 2 other reviewers |
| 13 | The purposeful selection of factors to include in the regression analysis (based on univariate analysis) might have excluded some important factors from the multivariate regression model. It would be good to discuss these in the discussion. | Thank you for the comment. We acknowledge that selecting variables based on univariate significance may exclude potentially important factors. Given the exploratory nature of our study and the sample size considerations, we used this approach to minimise overfitting.  However, we recognise its limitations and have now noted this in the discussion (page 16) |
| 14 | Moreover, lines 289-290, interpretation of non-association should be phrased cautiously, as these results are from univariate analysis only / or to be specified that this comes from univariate analysis only. | Thank you for the comment. We agree that non-significant findings, particularly those derived from univariate analysis, should be interpreted with caution. We have revised the manuscript to clarify that these results are based on univariate comparisons and may not account for confounding factors. Additionally, the retrospective design and small subgroup sizes further limit the strength of these conclusions. Future studies with prospective designs and larger cohorts are needed to confirm these patterns. |
| 15 | Also, in Table 4, for variables with more than 2 categories, the significance at variable level should also be presented (i.e. for “severity of TBI” and for “types of behavior issue”), moreover, presenting the CI in Table 4 would be useful. | We have addressed this and add the CI too |
| 16 | Recommendation for a coordinated MDT is indeed relevant, however: in line 347, cognitive, emotional and behavioral challenges are mentioned, when referring to recovery, while skipping the physical challenges/limitations commonly associated with TBI (and also included in the GOS-E, used as primary outcome in this study). | Thank you for highlighting this.  We have added the physical limitations as common challenges associated in TBI and should be addressed in the coordinated MDT |
| 17 | Moreover, this last part of the discussion would benefit some restructuring in terms of chronology, as discharge and follow-up needs are first mentioned, then jumping back to inpatient care, which might be kind of confusing for the reader. | We have re-written the discussion to make it more structured |
| 18 | The conclusion in the abstract (focusing on age-differences) differ a lot for the main conclusion (focusing on rehab strategies). It might be good to harmonize, moreover the link with this study findings are not direct. | We have edited the conclusion both in the abstract and in the text (under conclusion) to make it more align with the outcome of this study |

Reviewer #3

|  | Comments | Action |
| --- | --- | --- |
| 1. | The title of the manuscript is quite broad, and issues discussed in the paper are specific to Malaysia and Southeast Asia. It is suggested to add "in Malaysia" to the title somewhere to specify the region that was included in this study. | “Malaysia” has been added to the title to make it more specific:  Outcomes of school-aged children following traumatic brain injury from road traffic accidents in Malaysia: a retrospective study |
| 2. | In the methods section, GOS-E Peds is described as being suitable for individuals up to 16 years of age. However, this study includes the evaluation of individuals over 16 years of age. The authors should consider a comment in the discussion surrounding this.  How does GOS-E Peds differ from GOS-E? Is there any anticipated effect with using the pediatric version of GOS-E compared to the adult version? | Thank you for this observation.  Although the GOS-E Peds is validated for use up to 16 years of age, there is a small subset of our cohort of more than 16 years old at the time of review that were evaluated using GOS-E Peds.  GOS‑E Peds maintains a structure that is highly comparable to the adult GOS‑E, while adding developmentally appropriate items for younger populations. (Beers et al., 2012): GOS‑E Peds scores strongly correlated with GOS scores which can be used for adults at both 3- and 6-months post-injury.  The main difference is the assessment focus:   - GOS-E focus on adult responsibilities & independence. Key questions revolve around: **return to work**, ability to manage finances, maintain social and leisure activities, relationships with family and friends, and independence in running a household. - GOS-E Peds focus on age-appropriate development and dependency.   Key questions revolve around: **Return to school and academic performance**, ability to keep up with peers, play and socialize appropriately, behavior, and the extra burden of care placed on the family.  Given that the GOS-E Peds focuses on age-appropriate functional domains such as school reintegration, we felt it remained relevant and clinically meaningful for these older adolescents since they are still in the school-going age.  This approach was also chosen to maintain consistency in outcome measurement across the cohort. Nonetheless, this may introduce minor classification bias, and future studies may consider comparing both the paediatric and adult versions (GOS-E Peds vs. GOS-E) in transitional age groups.  We have added this explanation in the method section and add discussion to clarify any potential implications. |
| 3. | Return to school and academic performance data was not adequately described throughout the paper, and the statistics for these were not included in any table. For example, lines 195-196 indicate that children experienced "academic difficulties such as declining performance." What are other examples of academic difficulties? | The examples of the academic difficulties have been written in the discussion section. We presented them in general and not in specific tables according to the different age groups |
| 4. | In the discussion section, it would be great to discuss reference 14 in a bit more detail and compare the findings between children (this study) and adults (ref 14). Reference 14 was cited as a precursor to this paper, and as such many methods are similar offering a great opportunity to compare adult and pediatric populations. | This has been added in discussion |
| 5. | Are there additional studies the authors could reference to compare their data to regarding occurrence of pediatric TBI due to RTA and functional outcomes post-pediatric TBI in other parts of the world? | We have added a few studies from other parts of the world and added in the discussion |
| 6. | The authors should consider rewriting the conclusions section of the manuscript to reflect the conclusions drawn by the analysis that was performed in the study. However the conclusion section of the abstract section first stated that "No significant differences in functional outcomes were observed across age groups. The findings in this study underscore the need for age-targeted road safety education, injury prevention strategies..." It is a bit confusing to not have statistical difference across age groups and then advocate for age-targeted interventions. Consider comment regarding comparison to Ref. 14 in the discussion section, and revise the conclusions accordingly. | Thank you for the suggestion.  The conclusion for both abstract and in the manuscript have been re-written to reflect the study findings and reduce the confusion made earlier |
| 7. | The records from which the statistics presented in the study were assembled are not available for review from the reader, likely due to ethical concerns surrounding sharing individual personal medical records, especially those of minors. This should be addressed in the Data Availability Statement. | This has been addressed in the Data Availability Statement |
| 8. | Minor Comments:  Line 28: Suggest to add "in Malaysia" to end of sentence  Line 29: Change to "...study analysed medical records of 542 children..."  Line 30: Change "sociodemographic" to "sociodemographics"  Line 31: Change "The reports were grouped..." to "The subjects were grouped..." or something of the like.  Line 53-54: "RTAs represent a significant public health..." public health concern? issue? problem? Missing a word there. "...in low- and middle..." Delete the "the"  Line 56: Delete the "were"  Line 59: "...19 years, who were predominantly male."  Line 71: Replace "was" with "were"  Line 72-73: "...there have been no other published studies with Malaysian subjects since that time."  Line 74: "as a result of a RTA"  Line 83: Delete "the," instead it should read "examination of specialist medical reports"  Line 85-86: "medical insurance claimant purposes. The data were accessed..."  Line 88-89: "...focused on an adult population dataset."  Line 91: "...time of injury, who sustained a TBI due to a RTA, and had no prior TBIs. Patients with..."  Line 96: "time of injury, time since the injury..."  Line 141: "The proportion of missing data in this study was 0.29% with data missing..."  Line 151: Instead of "participants" consider using "subjects."  Line 152: "Reports for 542 individuals were included..."  Line 153: "...highest number of participants was the older..."  Line 162: "observed in young children"  Line 165-166: "The majority of the motorcycle-related cases..."  Line 170: "...is 16 years. In this study..."  Line 183: 20% should be 21%. Could also reference Figure 1 here.  Line 191: "with 8.6% on anti-epileptic medications."  Line 192-193: Does "mixed symptoms" indicate that an individual experienced multiple different behavioral issues? Please clarify.  Line 206-208: Could reference Figure 1 here. Should be 14.8% on line 207 to be consistent with digits on other percentages in this sentence.  Line 215: "...with 6% on anti-epileptic..."  Line 235: Could reference Table 3 here.  Line 240: The text indicated 66.8% requiring targeted interventions, but Table 3 indicates 58.8%. Appears that 58.8% is the correct number, please check.  Line 243-244: Text indicates "prescribed anti-epileptic medications," Tables 1-3 indicate anti-epileptic medication use. Prescribed anti-epileptic medication may be a better descriptor for Tables, unless there is documentation that these medications were in fact taken, not just prescribed.  Line 246: Text states "neither studying nor working." Tables state "not in school." Please fix tables to indicate the group is neither in school nor working.  Line 253: "There are eight factors that were significantly correlated with functional outcome..."  Line 277: "...aligns with previous research in Malaysia..."  Line 319: "...with a substantial proportion (75%) involving older adolescents."  Line 322: "...motorcycles are a widely used..." | All of these have been corrected.  Some of the sentences have been deleted due to the major changes in the content and arrangement of the discussion section after incorporating all the reviewers’ comments and suggestions.  Figure 1 has been referenced here  Yes, we have re-worded it  Figure 1 has been referenced here  66.8% reported in the text is in relation to 295 cases that sustained concomitant injury. In which, 197 (66.8%) requiring intervention. This is different from the percentage in the Table |
| 9. | Tables/Figure general - Motorcycle is used in text while motorbike is used in tables/figure. Please choose one term and be consistent throughout manuscript;  . | These have been corrected |
|  | Also Study/Work Status is assumed to be pre-injury, but this is not explicitly stated in Tables 2 and 3 as it is in Table 1. |  |
|  | Why is there a column with numbers on the left side of Table 3? |  |
|  | Table 4: "Tracheostomy (No)" should be italicized |  |
