# Supplementary material for: Outcomes of school-aged children following traumatic brain injury from road traffic accidents in Malaysia: A retrospective study
Source: PLoS One. 2026 Jun 16;21(6):e0351136. doi: 10.1371/journal.pone.0351136 (PMC13271509; doi:10.1371/journal.pone.0351136)
Supplement: Attachment — Submitted filename: Response_to_Reviewers_auresp_2.docx [file pone.0351136.s003.docx]

**Response to Reviewers (PLoS One)**

**PONE-D-25-33135R1**

**Outcomes of school-aged children following traumatic brain injury from road traffic accidents in Malaysia: a retrospective study**

**Dear Editor,**

|  | Comments | Action |
| --- | --- | --- |
| 1. | Improve the tables to be easier to read.  I find the tables difficult to learn; please add the unit (% or mean +/- SD in the lines. Several cells content only the number (with no rates); please be consistent and detail the percentage or the SD in all cells. All rates should be with a decimal to be consistent; some of them in the text are without decimals | We have revised all tables for consistency. Each variable now explicitly states units (e.g., percentages as “n (%)”, means with “± SD”), and all percentage values now include a single decimal point to improve readability. Rows with missing values or ambiguous formatting were clarified. A clearer column structure has been applied, and redundant or empty rows removed where possible.  All rates in the text have been added decimals when comparing values, to be consistent |
| 2. | Modify the manuscript following the reviewers' comments | All the comments have been addressed as explained in the subsequent sections |

Reviewer #3

|  | Comments |  |
| --- | --- | --- |
| 1. | The Introduction stated that very young children under 6 y/o experience poorer functional recovery (according to refs. 6-8), but data in this study suggests younger children (ages 7-9 years) had highest rate of recovery. Could you place this into context in the discussion? | We have added a discussion of the findings, which suggest that younger children (ages 7–9 years) had the highest rate of recovery (although not statistically significant). This contrasts with the statement in the Introduction that very young children (under 6 years old) experience poorer functional recovery (according to refs. 6–8). |
|  | Line 177: 542 reports for 542 subjects or 542 reports, but some subjects had multiple reports? This also came up earlier in the manuscript. Please be sure to clarify throughout the manuscript that there were 542 subjects. | We thank the reviewer for highlighting this point. We confirm that each subject had one medical report prepared by a senior consultant, who received referrals from across Malaysia. These reports were generated for insurance claim purposes, and no subject had more than one report. We have clarified this in the Methods section and ensured consistency throughout the manuscript. |
|  | Lines 220-224: There seems to be a difference between multiple injuries and concomitant injuries. Could you please clarify what this difference is throughout the manuscript? | “Multiple injuries” was written to explain that the subject has more than two concomitant injuries simultaneously. We have cleared this confusion and rephrased “multiple injuries” with “concomitant injuries affecting more than two body regions”. |
|  | Table 2 and Table 3: The percentages in parentheses are confusing. To add to 100% the reader needs to look vertically for the "n" column, then horizontally between the good and poor outcome columns. Maybe a thicker or double vertical line between the n column and the good outcome column would help? | For both Table 2 and Table 3:  We have added a thicker vertical line between the n column and the good outcome column |
|  | The rest of this review is limited to minor comments for clarity as follows:  Line 31: “the subjects reports…” should have an apostrophe - “the subjects’ reports” | This has been corrected |
|  | Line 57: "common cause" should be "common causes" | This has been corrected |
|  | Line 58: define LMIC here, then in the next sentence (line 59 where low- and middle-income countries is spelled out) use the abbreviation: “…death and disability in low- and middle-income countries (LMICs). 1,2 RTAs represent a significant public health concern in LMICs, including Malaysia.” | This has been corrected |
|  | Line 61-62: change to “…sustained brain injuries, with 95.4% of those cases attributed to RTAs.” or “…sustained brain injuries, and 95.4% of those cases were attributed to RTAs.” | This has been corrected |
|  | Line 62: “Furthermore, motorcycle accidents accounted for…” | This has been corrected |
|  | Line 67: “…differently than adults. This is largely…” | This has been corrected |
|  | Line 155: Missing a space, “category 1 or 2, indicating” | This has been corrected |
|  | Line 162: “The proportion of missing data values in this study was 0.29%...” | This has been corrected |
|  | Line 168: “…in which the forward stepwise likelihood ratio (LR) method was applied.” | This has been corrected |
|  | Line 169: “…performed for the adolescent group…” | This has been corrected |
|  | Line 173: “…final sample, which is presented in the results.” | This has been corrected |
|  | Line 176: table 1 -> Table 1 (capitalize Table) | This has been corrected |
|  | Line 197: “most common mode of injury in this study” | This has been corrected |
|  | Line 252: “with 6% on anti-epileptic medications.” | This has been corrected |
|  | Lin 282: “with 5.7% prescribed anti-epileptic medications.” | This has been corrected |
| 2. | Line 295: Table 3 (capitalize) | This has been corrected |
| 3. | Line 301: “…mild TBI patients...” | This has been corrected |
| 4. | Line 319: mechanism -> mode | This has been corrected |
| 5. | Line 345: “Greater TBI severity…” | This has been corrected |
| 6. | Line 392: “discontinued school entirely” | This has been corrected |
| 7. | Lines 415-416: “motorcycle-related TBIs are common among school-aged children in Malaysia, although there were...” | This has been corrected |

Reviewer #4

|  | Comments | Action |
| --- | --- | --- |
| 1. | Add at the end of discussion section a separate heading - Limitations of the study in bold. Under it those observations that were not fully complied, being a retrospective analysis and other reasons, can be mentioned. Statistical analysis related issues may also be added as well as choice of tool used and which areas could not be assessed as post injury outcome. | A new section titled Limitations of the study has been added to the end of the Discussion, outlining retrospective design, missing data, tool limitations, and lack of prehospital variables. Most of these limitations have been written in the manuscript earlier, but without a separate heading. Therefore, we pulled them to be written in this separate heading. |
