# Supplementary material for: Outcomes of school-aged children following traumatic brain injury from road traffic accidents in Malaysia: A retrospective study
Source: PLoS One. 2026 Jun 16;21(6):e0351136. doi: 10.1371/journal.pone.0351136 (PMC13271509; doi:10.1371/journal.pone.0351136)
Supplement: Peer review history

# Peer review: Decision Letter 0

**DOI:** 10.1371/journal.pone.0351136.r001

**Authors:** Olfa Chakroun-Walha

23 Jul 2025

PONE-D-25-33135

Outcomes of school-aged children following traumatic brain injury from road traffic accidents: a retrospective study

PLOS ONE

Dear Dr. Mazlan,

Thank you for submitting your manuscript to PLOS ONE. After careful consideration, we feel that it has merit but does not fully meet PLOS ONE’s publication criteria as it currently stands. Therefore, we invite you to submit a revised version of the manuscript that addresses the points raised during the review process.

==============================

**ACADEMIC EDITOR: we invite to re-write your manuscript following the reviewers' comments.**

**The methods section needs a lot of improvements and the discussion lacks of comparison with other studies conducted in similar settings. your manuscript can be considered if the methods and the discussion are reformulated;**

==============================

Please submit your revised manuscript by Sep 06 2025 11:59PM. If you will need more time than this to complete your revisions, please reply to this message or contact the journal office at plosone@plos.org. When you're ready to submit your revision, log on to https://www.editorialmanager.com/pone/ and select the 'Submissions Needing Revision' folder to locate your manuscript file.

Please include the following items when submitting your revised manuscript:

- A rebuttal letter that responds to each point raised by the academic editor and reviewer(s). You should upload this letter as a separate file labeled 'Response to Reviewers'.
- A marked-up copy of your manuscript that highlights changes made to the original version. You should upload this as a separate file labeled 'Revised Manuscript with Track Changes'.
- An unmarked version of your revised paper without tracked changes. You should upload this as a separate file labeled 'Manuscript'.

If you would like to make changes to your financial disclosure, please include your updated statement in your cover letter. Guidelines for resubmitting your figure files are available below the reviewer comments at the end of this letter.

If applicable, we recommend that you deposit your laboratory protocols in protocols.io to enhance the reproducibility of your results. Protocols.io assigns your protocol its own identifier (DOI) so that it can be cited independently in the future. For instructions see: https://journals.plos.org/plosone/s/submission-guidelines#loc-laboratory-protocols. Additionally, PLOS ONE offers an option for publishing peer-reviewed Lab Protocol articles, which describe protocols hosted on protocols.io. Read more information on sharing protocols at https://plos.org/protocols?utm_medium=editorial-email&utm_source=authorletters&utm_campaign=protocols.

We look forward to receiving your revised manuscript.

Kind regards,

Olfa Chakroun-Walha

Academic Editor

PLOS ONE

Journal Requirements:

When submitting your revision, we need you to address these additional requirements.

1. Please ensure that your manuscript meets PLOS ONE's style requirements, including those for file naming. The PLOS ONE style templates can be found at https://journals.plos.org/plosone/s/file?id=wjVg/PLOSOne_formatting_sample_main_body.pdf and https://journals.plos.org/plosone/s/file?id=ba62/PLOSOne_formatting_sample_title_authors_affiliations.pdf

2. Please provide additional details regarding participant consent. In the ethics statement in the Methods and online submission information, please ensure that you have specified (1) whether consent was informed and (2) what type you obtained (for instance, written or verbal, and if verbal, how it was documented and witnessed). If your study included minors, state whether you obtained consent from parents or guardians. If the need for consent was waived by the ethics committee, please include this information.

If you are reporting a retrospective study of medical records or archived samples, please ensure that you have discussed whether all data were fully anonymized before you accessed them and/or whether the IRB or ethics committee waived the requirement for informed consent. If patients provided informed written consent to have data from their medical records used in research, please include this information

3. We note that you have indicated that there are restrictions to data sharing for this study. For studies involving human research participant data or other sensitive data, we encourage authors to share de-identified or anonymized data. However, when data cannot be publicly shared for ethical reasons, we allow authors to make their data sets available upon request. For information on unacceptable data access restrictions, please see http://journals.plos.org/plosone/s/data-availability#loc-unacceptable-data-access-restrictions.

Before we proceed with your manuscript, please address the following prompts:

a) If there are ethical or legal restrictions on sharing a de-identified data set, please explain them in detail (e.g., data contain potentially identifying or sensitive patient information, data are owned by a third-party organization, etc.) and who has imposed them (e.g., a Research Ethics Committee or Institutional Review Board, etc.). Please also provide contact information for a data access committee, ethics committee, or other institutional body to which data requests may be sent.

b) If there are no restrictions, please upload the minimal anonymized data set necessary to replicate your study findings to a stable, public repository and provide us with the relevant URLs, DOIs, or accession numbers. Please see http://www.bmj.com/content/340/bmj.c181.long for guidelines on how to de-identify and prepare clinical data for publication. For a list of recommended repositories, please see https://journals.plos.org/plosone/s/recommended-repositories. You also have the option of uploading the data as Supporting Information files, but we would recommend depositing data directly to a data repository if possible.

Please update your Data Availability statement in the submission form accordingly.

4. If the reviewer comments include a recommendation to cite specific previously published works, please review and evaluate these publications to determine whether they are relevant and should be cited. There is no requirement to cite these works unless the editor has indicated otherwise.

Additional Editor Comments :

This is an interesting retrospective research focusing on outcomes of TBI in children and adolescents in a LMIC

the manuscript is well written, the results are well presented and the study is conducted in a region which is not very presented in the literature

However, several major concerns need to be improved

The title is very broad, a more specific title, stressing on the setting, is encouraged

The methods section needs a lot of changes: the details concerning how the sample size calculation was done, the steps and variables of the multivariate statistical analysis

The manuscript can be considered if it's rewritten following the reviewers comments, namely with more focus on the regional features of tha study population and the potentially impacts on the local policies.

In the results section, why the prehospital variables haven't been mentioned,

The discussion section lucks the comparison with local and regional findings, the results presented was widely studied in the literature but notnin a such setting

The conclusion is not adapted to the results presented here

[Note: HTML markup is below. Please do not edit.]

Reviewers' comments:

Reviewer's Responses to Questions

-->Comments to the Author

1. Is the manuscript technically sound, and do the data support the conclusions?

The manuscript must describe a technically sound piece of scientific research with data that supports the conclusions. Experiments must have been conducted rigorously, with appropriate controls, replication, and sample sizes. The conclusions must be drawn appropriately based on the data presented.

Reviewer #1: Partly

Reviewer #2: Partly

Reviewer #3: Partly

**********

2. Has the statistical analysis been performed appropriately and rigorously?

Reviewer #1: Yes

Reviewer #2: Yes

Reviewer #3: I Don't Know

**********

3. Have the authors made all data underlying the findings in their manuscript fully available?

The PLOS Data policy requires authors to make all data underlying the findings described in their manuscript fully available without restriction, with rare exception (please refer to the Data Availability Statement in the manuscript PDF file). The data should be provided as part of the manuscript or its supporting information, or deposited to a public repository. For example, in addition to summary statistics, the data points behind means, medians and variance measures should be available. If there are restrictions on publicly sharing data—e.g. participant privacy or use of data from a third party—those must be specified.

Reviewer #1: Yes

Reviewer #2: No

Reviewer #3: No

**********

4. Is the manuscript presented in an intelligible fashion and written in standard English?

PLOS ONE does not copyedit accepted manuscripts, so the language in submitted articles must be clear, correct, and unambiguous. Any typographical or grammatical errors should be corrected at revision, so please note any specific errors here.

Reviewer #1: Yes

Reviewer #2: Yes

Reviewer #3: Yes

**********

5. Review Comments to the Author

Please use the space provided to explain your answers to the questions above. You may also include additional comments for the author, including concerns about dual publication, research ethics, or publication ethics. (Please upload your review as an attachment if it exceeds 20,000 characters)

Reviewer #1: Title: Outcomes of School-aged Children Following Traumatic Brain Injury From Road Traffic Accidents: A Retrospective Study

This paper presents the results of statistical and logistic regression analysis of children who have suffered traumatic brain injury (TBI) following their involvement in a road traffic accident (RTA). The paper presents original research, and results have not been published elsewhere. The methodology employed in the study is sound, that is, univariate analysis and logistic regression. Appropriate proxy measures of TBI severity and functional outcomes have been used. The authors appropriately recognise limitations due to sample sizes.

Overall the paper is well constructed and makes some good observations. However, I feel the conclusions are very high level and somewhat obvious (obvious may not be the best word here). For instance, I feel the conclusion that provision of "age-targeted road safety education" is not a direct outcome of this study but rather the study findings support the continuance of existing programs that do this already.

The paper is well written and well formatted. It was understandable and easy to read. The research meets applicable ethics standards and is appropriately reported.

There are some questions around the data. For instance, the proportion of Motorcycles as the vehicle involved in the RTA resulting in TBI seems excessive. Does this reflect the proportion of motorcycles on the roads? I would also question why pre-hospital observations of accident scene, vital signs, interventions, and then more in-hospital diagnosis and procedures were not used as part of the study. The authors have included the occurence of tracheostomy, ICU admission, neurosurgical intervention and intervention for concomitant injury as factors. However, these are simply binary values. As only tracheostomy appeared as a significant factor, it may have been worthwhile considering more granular representations of other variables. For instance ICU Length of Stay. Inclusion of these additional attributes may shed more light on factors contributing to both TBI level and functional recovery.

Nevertheless, the study provides some very useful data about the makeup of different groups of school age children, RTA types, proportion of severity of TBI, and lastly functional outcomes across the different groups.

However, my main concern is that I do not feel that this paper is a significant contribution to the body of work around RTA, TBI and functional outcomes. This has been extensively studied and written about with the conclusion that TBI severity resulting from RTA is a significant predictor of functional outcome. The novelty of the work lies, perhaps in the regional considerations.

Suggestions for the Authors

1. I feel that an avenue worth exploring would be to use the results of the study as a basis for predictive support required by individual RTA victims. For instance, given the socio-demographic, RTA, and TBI related factors, could a prediction of functional outcome be made, and hence back-to-school or return-to-work support program be recommended based on the anticipated functional outcome.

2. I feel that an additional line of investigation may have been pairwise analysis variables (e.g. correlation analysis). For instance, is neurosurgical intervention related to TBI severity?

3. I also feel that inclusion of more RTA factors may be useful. For instance, in the case of motorcycle accident, was the injured person rider or pillion passenger, was the injured person wearing a helmet, what was the other vehicle involved in the collision. In our work with RTAs we have found other factors such as vehicle rollover, ejection from the vehicle, fractures, injury to multiple body regions, etc. being predictive of injury severity. Pre-hospital factors are also relevant to injury severity, recovery and subsequent functional outcomes. Tracheostomy pre-hospital is one such indicator. Others include blood transfusion and CPR.

Reviewer #2: This manuscript addresses an evidence gap by documenting outcomes in children with TBI in a LMIC, which is largely underreported. The affiliation from both Surgery and Rehab departments is a plus, ensuring different professional perspectives. The sample size is also relatively large, differing from other studies in similar contexts. The use of routine data strengthens its transferability to practice in Malaysia and other LMICs. Moreover, the focus on return to education and related challenges is also worth mentioning as it really fosters links with future policy changes.

However, the manuscript would benefit from some major and minor revisions before its publication:

*Major revisions:

The data availability statement is no complete, as it states that “all relevant data are within the manuscript” while the manuscript only includes aggregated data. Further clarification might be needed on that regard.

The statistical analysis part also requires some attention.

-Firstly, there is no information on the sample size calculation, which doesn’t allow the reader to appreciate whether there is enough power for the analyses performed, especially the regression analysis.

-Also, the reason to run the analyses per subgroup instead of including age as a covariate is not clear, especially given the limited sample size in two of the groups.

-In the regression model run with older adolescents, the variables inclusion process is not clear/does not match what is explained in the methods section. Indeed, among the 8 factors significantly associated with the outcome in the univariate analysis (table 3), 5 did not seem to be included in the multivariate analysis (Table 4).

*Minor revisions:

-Specifying the context in the title would be good.

-In the introduction, reference for global data is relatively old and could benefit some updates. Moreover, references are sometimes placed at the end, sometimes in the middle of sentences (e.g. line 71), which would be good to harmonize.

-The use of routine data is an asset, however it carries also some risks, including selection bias. It would be good to discuss the implications of such data source in the discussion.

-The definition and source of information for the variable on “complication” needs some clarification and consistency between methods and results.

-The follow-up period was highly heterogenous (20M +/- 15M). How was this tackled in the analysis? Any sensitivity analyses done to check whether time to injury (at report) was associated with level of recovery? Which implications might it have on findings?

-Given the research questions, having the table 1 presented by age groups, including testing differences in terms of sociodemographic and injury characteristics would help the reader to capture those differences and read the results accordingly.

-In Discussion, the findings are not always compared to existing literature (especially in first third of discussion), which weakens interpretation (e.g non significance of given factors, return to school and experienced difficulties,..)

-The purposeful selection of factors to include in the regression analysis (based on univariate analysis) might have excluded some important factors from the multivariate regression model. It would be good to discuss these in the discussion.

Moreover, lines 289-290, interpretation of non association should be phrased cautiously, as these results are from univariate analysis only / or to be specified that this comes from univariate analysis only.

-Also, in Table 4, for variables with more than 2 categories, the significance at variaible level should also be presented (i.e. for “severity of TBI” and for “types of behavior issue”), moreover, presenting the CI in Table 4 would be useful.

-Recommendation for a coordinated MDT is indeed relevant, however: in line 347, cognitive, emotional and behavioral challenges are mentioned, when referring to recovery, while skipping the physical challenges/limitations commonly associated with TBI (and also included in the GOS-E, used as primary outcome in this study). Moreover, this last part of the discussion would benefit some restructuring in terms of chronology, as discharge and follow-up needs are first mentioned, then jumping back to inpatient care, which might be kind of confusing for the reader.

-The conclusion in the abstract (focusing on age-differences) differ a lot for the main conclusion (focusing on rehab strategies). It might be good to harmonize, moreover the link with this study findings are not direct.

Reviewer #3: Overall this manuscript highlights an important area of public health concern in Malaysia and greater Southeast Asia: pediatric TBI due to road traffic accidents. Analyses such as this are convincing when governments/companies consider things like regulation and development of better protective equipment, and as such it is important studies like this are published for public consumption. However, it seems to this reviewer that the connections between the statistical analysis presented and the suggestion for preventative and rehabilitative resources is not made as strongly as it could be. With some additional review and synthesis, this study would be worthy of publication.

Major Comments:

The title of the manuscript is quite broad, and issues discussed in the paper are specific to Malaysia and Southeast Asia. In other parts of the world, motorcycles/motorbikes are far less common, and children riding (either as operator or passenger) is even more rare. It is suggested to add "in Malaysia" to the title somewhere to specify the region that was included in this study.

In the methods section, GOS-E Peds is described as being suitable for individuals up to 16 years of age. However, this study includes the evaluation of individuals over 16 years of age. The authors should consider a comment in the discussion surrounding this. How does GOS-E Peds differ from GOS-E? Is there any anticipated effect with using the pediatric version of GOS-E compared to the adult version?

Return to school and academic performance data was not adequately described throughout the paper, and the statistics for these were not included in any table. For example, lines 195-196 indicate that children experienced "academic difficulties such as declining performance." What are other examples of academic difficulties?

In the discussion section, it would be great to discuss reference 14 in a bit more detail and compare the findings between children (this study) and adults (ref 14). Reference 14 was cited as a precursor to this paper, and as such many methods are similar offering a great opportunity to compare adult and pediatric populations.

The discussion section seems to highlight gaps in prevention resources and rehabilitation resources after pediatric TBI in Malaysia and other parts of Southeast Asia. Are there additional studies the authors could reference to compare their data to regarding occurrence of pediatric TBI due to RTA and functional outcomes post-pediatric TBI in other parts of the world? This would bolster the need for the prevention and rehabilitation resources the manuscript currently promotes.

The conclusion section of the abstract includes a better summary of the data presented than the conclusion section of the manuscript itself. The conclusion section of the manuscript seems to highlight steps that need to be taken to improve functional outcomes for children following TBI, but does not highlight any conclusions from the analysis that was done in this study. The authors should consider rewriting the conclusions section of the manuscript to reflect the conclusions drawn by the analysis that was performed in the study. However the conclusion section of the abstract section first stated that "No significant differences in functional outcomes were observed across age groups. The findings in this study underscore the need for age-targeted road safety education, injury prevention strategies..." It is a bit confusing to not have statistical difference across age groups and then advocate for age-targeted interventions. Consider comment regarding comparison to Ref. 14 in the discussion section, and revise the conclusions accordingly.

The records from which the statistics presented in the study were assembled are not available for review from the reader, likely due to ethical concerns surrounding sharing individual personal medical records, especially those of minors. This should be addressed in the Data Availability Statement.

Minor Comments:

Additional line-by-line minor comments/suggestions are below. The language at times is a bit unclear, and the line-by-line comments in the attachment are meant to address some of this issue.

Line 28: Suggest to add "in Malaysia" to end of sentence

Line 29: Change to "...study analysed medical records of 542 children..."

Line 30: Change "sociodemographic" to "sociodemographics"

Line 31: Change "The reports were grouped..." to "The subjects were grouped..." or something of the like.

Line 53-54: "RTAs represent a significant public health..." public health concern? issue? problem? Missing a word there. "...in low- and middle..." Delete the "the"

Line 56: Delete the "were"

Line 59: "...19 years, who were predominantly male."

Line 71: Replace "was" with "were"

Line 72-73: "...there have been no other published studies with Malaysian subjects since that time."

Line 74: "as a result of a RTA"

Line 83: Delete "the," instead it should read "examination of specialist medical reports"

Line 85-86: "medical insurance claimant purposes. The data were accessed..."

Line 88-89: "...focused on an adult population dataset."

Line 91: "...time of injury, who sustained a TBI due to a RTA, and had no prior TBIs. Patients with..."

Line 96: "time of injury, time since the injury..."

Line 141: "The proportion of missing data in this study was 0.29% with data missing..."

Line 151: Instead of "participants" consider using "subjects."

Line 152: "Reports for 542 individuals were included..."

Line 153: "...highest number of participants was the older..."

Line 162: "observed in young children"

Line 165-166: "The majority of the motorcycle-related cases..."

Line 170: "...is 16 years. In this study..."

Line 183: 20% should be 21%. Could also reference Figure 1 here.

Line 191: "with 8.6% on anti-epileptic medications."

Line 192-193: Does "mixed symptoms" indicate that an individual experienced multiple different behavioral issues? Please clarify.

Line 206-208: Could reference Figure 1 here. Should be 14.8% on line 207 to be consistent with digits on other percentages in this sentence.

Line 215: "...with 6% on anti-epileptic..."

Line 235: Could reference Table 3 here.

Line 240: The text indicated 66.8% requiring targeted interventions, but Table 3 indicates 58.8%. Appears that 58.8% is the correct number, please check.

Line 243-244: Text indicates "prescribed anti-epileptic medications," Tables 1-3 indicate anti-epileptic medication use. Prescribed anti-epileptic medication may be a better descriptor for Tables, unless there is documentation that these medications were in fact taken, not just prescribed.

Line 246: Text states "neither studying nor working." Tables state "not in school." Please fix tables to indicate the group is neither in school nor working.

Line 253: "There are eight factors that were significantly correlated with functional outcome..."

Line 277: "...aligns with previous research in Malaysia..."

Line 319: "...with a substantial proportion (75%) involving older adolescents."

Line 322: "...motorcycles are a widely used..."

Tables/Figure general - Motorcycle is used in text while motorbike is used in tables/figure. Please choose one term and be consistent throughout manuscript; Also Study/Work Status is assumed to be pre-injury, but this is not explicitly stated in Tables 2 and 3 as it is in Table 1. Why is there a column with numbers on the left side of Table 3?

Table 4: "Tracheostomy (No)" should be italicized.

**********

6. PLOS authors have the option to publish the peer review history of their article (what does this mean?). If published, this will include your full peer review and any attached files.

If you choose “no”, your identity will remain anonymous but your review may still be made public.

Do you want your identity to be public for this peer review? For information about this choice, including consent withdrawal, please see our Privacy Policy.

Reviewer #1: Yes: Robert Andrews

Reviewer #2: No

Reviewer #3: No

**********

[NOTE: If reviewer comments were submitted as an attachment file, they will be attached to this email and accessible via the submission site. Please log into your account, locate the manuscript record, and check for the action link "View Attachments". If this link does not appear, there are no attachment files.]

While revising your submission, please upload your figure files to the Preflight Analysis and Conversion Engine (PACE) digital diagnostic tool, https://pacev2.apexcovantage.com/. PACE helps ensure that figures meet PLOS requirements. To use PACE, you must first register as a user. Registration is free. Then, login and navigate to the UPLOAD tab, where you will find detailed instructions on how to use the tool. If you encounter any issues or have any questions when using PACE, please email PLOS at figures@plos.org. Please note that Supporting Information files do not need this step.

# Author comment: Author response to Decision Letter 1

**DOI:** 10.1371/journal.pone.0351136.r002

18 Oct 2025

Dear Editor & reviewers, we have addressed all the comments and have uploaded the document as "Respond to Reviewers" file in the attached file section.

Attachment Submitted filename: Response to Reviewers.docx

# Peer review: Decision Letter 1

**DOI:** 10.1371/journal.pone.0351136.r003

**Authors:** Olfa Chakroun-Walha

22 Dec 2025

PONE-D-25-33135R1

Outcomes of school-aged children following traumatic brain injury from road traffic accidents in Malaysia: a retrospective study

PLOS One

Dear Dr. Mazlan,

Thank you for submitting your manuscript to PLOS ONE. After careful consideration, we feel that it has merit but does not fully meet PLOS ONE’s publication criteria as it currently stands. Therefore, we invite you to submit a revised version of the manuscript that addresses the points raised during the review process.

==============================

**ACADEMIC EDITOR:**

- Improve the tables to be easier to read
- Modify the manuscript following the reviewers' comments

-->==============================

Please submit your revised manuscript by Feb 05 2026 11:59PM. If you will need more time than this to complete your revisions, please reply to this message or contact the journal office at plosone@plos.org. When you're ready to submit your revision, log on to https://www.editorialmanager.com/pone/ and select the 'Submissions Needing Revision' folder to locate your manuscript file.

Please include the following items when submitting your revised manuscript:

- A letter that responds to each point raised by the academic editor and reviewer(s). You should upload this letter as a separate file labeled 'Response to Reviewers'.
- A marked-up copy of your manuscript that highlights changes made to the original version. You should upload this as a separate file labeled 'Revised Manuscript with Track Changes'.
- An unmarked version of your revised paper without tracked changes. You should upload this as a separate file labeled 'Manuscript'.

-->If you would like to make changes to your financial disclosure, please include your updated statement in your cover letter. Guidelines for resubmitting your figure files are available below the reviewer comments at the end of this letter.

If applicable, we recommend that you deposit your laboratory protocols in protocols.io to enhance the reproducibility of your results. Protocols.io assigns your protocol its own identifier (DOI) so that it can be cited independently in the future. For instructions see: https://journals.plos.org/plosone/s/submission-guidelines#loc-laboratory-protocols. Additionally, PLOS ONE offers an option for publishing peer-reviewed Lab Protocol articles, which describe protocols hosted on protocols.io. Read more information on sharing protocols at https://plos.org/protocols?utm_medium=editorial-email&utm_source=authorletters&utm_campaign=protocols.

We look forward to receiving your revised manuscript.

Kind regards,

Olfa Chakroun-Walha

Academic Editor

PLOS One

Journal Requirements:

If the reviewer comments include a recommendation to cite specific previously published works, please review and evaluate these publications to determine whether they are relevant and should be cited. There is no requirement to cite these works unless the editor has indicated otherwise.

Please review your reference list to ensure that it is complete and correct. If you have cited papers that have been retracted, please include the rationale for doing so in the manuscript text, or remove these references and replace them with relevant current references. Any changes to the reference list should be mentioned in the rebuttal letter that accompanies your revised manuscript. If you need to cite a retracted article, indicate the article’s retracted status in the References list and also include a citation and full reference for the retraction notice.

Additional Editor Comments:

The comments have been answered

I find the tables difficult to learn; please add the unit (% or mean +/- SD in the lines. several cells content only the number (with no rates); please be consistent and detail the percentage or the SD in all cells

All rates should be with a decimal to be consistent; some of them in the text are without decimals

[Note: HTML markup is below. Please do not edit.]

Reviewers' comments:

Reviewer's Responses to Questions

**Comments to the Author**

1. If the authors have adequately addressed your comments raised in a previous round of review and you feel that this manuscript is now acceptable for publication, you may indicate that here to bypass the “Comments to the Author” section, enter your conflict of interest statement in the “Confidential to Editor” section, and submit your "Accept" recommendation.

Reviewer #3: (No Response)

Reviewer #4: All comments have been addressed

**********

2. Is the manuscript technically sound, and do the data support the conclusions?

The manuscript must describe a technically sound piece of scientific research with data that supports the conclusions. Experiments must have been conducted rigorously, with appropriate controls, replication, and sample sizes. The conclusions must be drawn appropriately based on the data presented.

Reviewer #3: Yes

Reviewer #4: Yes

**********

3. Has the statistical analysis been performed appropriately and rigorously?

Reviewer #3: Yes

Reviewer #4: I Don't Know

**********

4. Have the authors made all data underlying the findings in their manuscript fully available?

The PLOS Data policy requires authors to make all data underlying the findings described in their manuscript fully available without restriction, with rare exception (please refer to the Data Availability Statement in the manuscript PDF file). The data should be provided as part of the manuscript or its supporting information, or deposited to a public repository. For example, in addition to summary statistics, the data points behind means, medians and variance measures should be available. If there are restrictions on publicly sharing data—e.g. participant privacy or use of data from a third party—those must be specified.

Reviewer #3: (No Response)

Reviewer #4: Yes

**********

5. Is the manuscript presented in an intelligible fashion and written in standard English?

PLOS ONE does not copyedit accepted manuscripts, so the language in submitted articles must be clear, correct, and unambiguous. Any typographical or grammatical errors should be corrected at revision, so please note any specific errors here.

Reviewer #3: Yes

Reviewer #4: Yes

**********

6. Review Comments to the Author

Please use the space provided to explain your answers to the questions above. You may also include additional comments for the author, including concerns about dual publication, research ethics, or publication ethics. (Please upload your review as an attachment if it exceeds 20,000 characters)

Reviewer #3: The updated version of this manuscript is much improved over the initial submission. There are just a few issues that should be addressed before acceptance:

The Introduction stated that very young children under 6 y/o experience poorer functional recovery (according to refs. 6-8), but data in this study suggests younger children (ages 7-9 years) had highest rate of recovery. Could you place this into context in the discussion?

Line 177: 542 reports for 542 subjects or 542 reports, but some subjects had multiple reports? This also came up earlier in the manuscript. Please be sure to clarify throughout the manuscript that there were 542 subjects.

Lines 220-224: There seems to be a difference between multiple injuries and concomitant injuries. Could you please clarify what this difference is throughout the manuscript?

Table 2 and Table 3: The percentages in parentheses are confusing. To add to 100% the reader needs to look vertically for the "n" column, then horizontally between the good and poor outcome columns. Maybe a thicker or double vertical line between the n column and the good outcome column would help?

The rest of this review is limited to minor comments for clarity as follows:

Line 31: “the subjects reports…” should have an apostrophe - “the subjects’ reports”

Line 57: "common cause" should be "common causes"

Line 58: define LMIC here, then in the next sentence (line 59 where low- and middle-income countries is spelled out) use the abbreviation: “…death and disability in low- and middle-income countries (LMICs). 1,2 RTAs represent a significant public health concern in LMICs, including Malaysia.”

Line 61-62: change to “…sustained brain injuries, with 95.4% of those cases attributed to RTAs.” or “…sustained brain injuries, and 95.4% of those cases were attributed to RTAs.”

Line 62: “Furthermore, motorcycle accidents accounted for…”

Line 67: “…differently than adults. This is largely…”

Line 155: Missing a space, “category 1 or 2, indicating”

Line 162: “The proportion of missing data values in this study was 0.29%...”

Line 168: “…in which the forward stepwise likelihood ratio (LR) method was applied.”

Line 169: “…performed for the adolescent group…”

Line 173: “…final sample, which is presented in the results.”

Line 176: table 1 -> Table 1 (capitalize Table)

Line 197: “most common mode of injury in this study”

Line 252: “with 6% on anti-epileptic medications.”

Lin 282: “with 5.7% prescribed anti-epileptic medications.”

Line 295: Table 3 (capitalize)

Line 301: “…mild TBI patients...”

Line 319: mechanism -> mode

Line 345: “Greater TBI severity…”

Line 392: “discontinued school entirely”

Lines 415-416: “motorcycle-related TBIs are common among school-aged children in Malaysia, although there were...”

Reviewer #4: Add at the end of discussion section a separate heading - Limitations of the study in bold. Under it those observations that were not fully complied, being a retrospective analysis and other reasons, can be mentioned. Statistical analysis related issues may also be added as well as choice of tool used and which areas could not be assessed as post injury outcome.

**********

7. PLOS authors have the option to publish the peer review history of their article (what does this mean?). If published, this will include your full peer review and any attached files.

If you choose “no”, your identity will remain anonymous but your review may still be made public.

**Do you want your identity to be public for this peer review?**   For information about this choice, including consent withdrawal, please see our Privacy Policy.

Reviewer #3: No

Reviewer #4: **Yes:**   JAMSHED AKHTAR

**********

[NOTE: If reviewer comments were submitted as an attachment file, they will be attached to this email and accessible via the submission site. Please log into your account, locate the manuscript record, and check for the action link "View Attachments". If this link does not appear, there are no attachment files.]

To ensure your figures meet our technical requirements, please review our figure guidelines: https://journals.plos.org/plosone/s/figures

You may also use PLOS’s free figure tool, NAAS, to help you prepare publication quality figures: https://journals.plos.org/plosone/s/figures#loc-tools-for-figure-preparation.

NAAS will assess whether your figures meet our technical requirements by comparing each figure against our figure specifications.

# Author comment: Author response to Decision Letter 2

**DOI:** 10.1371/journal.pone.0351136.r004

7 Jan 2026

Comments by Editor:

1. Improve the tables to be easier to read. I find the tables difficult to learn; please add the unit (% or mean +/- SD in the lines. Several cells content only the number (with no rates); please be consistent and detail the percentage or the SD in all cells. All rates should be with a decimal to be consistent; some of them in the text are without decimals

Reply: We have revised all tables for consistency. Each variable now explicitly states units (e.g., percentages as “n (%)”, means with “± SD”), and all percentage values now include a single decimal point to improve readability. Rows with missing values or ambiguous formatting were clarified. A clearer column structure has been applied, and redundant or empty rows removed where possible. All rates in the text have been added decimals when comparing values, to be consistent

2. Modify the manuscript following the reviewers' comments

Reply: All the comments have been addressed as explained in the subsequent sections

Comments by Reviewer #3:

1. The Introduction stated that very young children under 6 y/o experience poorer functional recovery (according to refs. 6-8), but data in this study suggests younger children (ages 7-9 years) had highest rate of recovery. Could you place this into context in the discussion?

Reply: We have added a discussion of the findings, which suggest that younger children (ages 7–9 years) had the highest rate of recovery (although not statistically significant). This contrasts with the statement in the Introduction that very young children.

2. Line 177: 542 reports for 542 subjects or 542 reports, but some subjects had multiple reports? This also came up earlier in the manuscript. Please be sure to clarify throughout the manuscript that there were 542 subjects.

Reply: We thank the reviewer for highlighting this point. We confirm that each subject had one medical report prepared by a senior consultant, who received referrals from across Malaysia. These reports were generated for insurance claim purposes, and no subject had more than one report. We have clarified this in the Methods section and ensured consistency throughout the manuscript.

3. Lines 220-224: There seems to be a difference between multiple injuries and concomitant injuries. Could you please clarify what this difference is throughout the manuscript?

Reply: “Multiple injuries” was written to explain that the subject has more than two concomitant injuries simultaneously. We have cleared this confusion and rephrased “multiple injuries” with “concomitant injuries affecting more than two body regions”.

4. Table 2 and Table 3: The percentages in parentheses are confusing. To add to 100% the reader needs to look vertically for the "n" column, then horizontally between the good and poor outcome columns. Maybe a thicker or double vertical line between the n column and the good outcome column would help?

Reply: For both Table 2 and Table 3:

We have added a thicker vertical line between the n column and the good outcome column

5. The rest of this review is limited to minor comments for clarity (typo errors, grammatical errors, etc)

Reply: All the mionr comments have been corrected accordingly in the text

Comment by Reviewer #4:

1. Add at the end of discussion section a separate heading - Limitations of the study in bold. Under it those observations that were not fully complied, being a retrospective analysis and other reasons, can be mentioned. Statistical analysis related issues may also be added as well as choice of tool used and which areas could not be assessed as post injury outcome.

Reply: A new section titled Limitations of the study has been added to the end of the Discussion, outlining retrospective design, missing data, tool limitations, and lack of prehospital variables. Most of these limitations have been written in the manuscript earlier, but without a separate heading. Therefore, we pulled them to be written in this separate heading.

Attachment Submitted filename: Response_to_Reviewers_auresp_2.docx

# Peer review: Decision Letter 2

**DOI:** 10.1371/journal.pone.0351136.r005

**Authors:** Nik Hisamuddin Nik Ab. Rahman

25 May 2026

Outcomes of school-aged children following traumatic brain injury from road traffic accidents in Malaysia: a retrospective study

PONE-D-25-33135R2

Dear Dr. Mazlan,

We’re pleased to inform you that your manuscript has been judged scientifically suitable for publication and will be formally accepted for publication once it meets all outstanding technical requirements.

Within one week, you’ll receive an e-mail detailing the required amendments. When these have been addressed, you’ll receive a formal acceptance letter and your manuscript will be scheduled for publication.

An invoice will be generated when your article is formally accepted. Please note, if your institution has a publishing partnership with PLOS and your article meets the relevant criteria, all or part of your publication costs will be covered. Please make sure your user information is up-to-date by logging into Editorial Manager at Editorial Manager® and clicking the ‘Update My Information' link at the top of the page. For questions related to billing, please contact billing support.

If your institution or institutions have a press office, please notify them about your upcoming paper to help maximize its impact. If they’ll be preparing press materials, please inform our press team as soon as possible -- no later than 48 hours after receiving the formal acceptance. Your manuscript will remain under strict press embargo until 2 pm Eastern Time on the date of publication. For more information, please contact onepress@plos.org.

Kind regards,

Nik Hisamuddin Nik Ab. Rahman

Academic Editor

PLOS One

Additional Editor Comments (optional):

Reviewers' comments:

Reviewer's Responses to Questions

**Comments to the Author**

1. If the authors have adequately addressed your comments raised in a previous round of review and you feel that this manuscript is now acceptable for publication, you may indicate that here to bypass the “Comments to the Author” section, enter your conflict of interest statement in the “Confidential to Editor” section, and submit your "Accept" recommendation.

Reviewer #3: All comments have been addressed

Reviewer #4: All comments have been addressed

Reviewer #5: All comments have been addressed

**********

2. Is the manuscript technically sound, and do the data support the conclusions?

The manuscript must describe a technically sound piece of scientific research with data that supports the conclusions. Experiments must have been conducted rigorously, with appropriate controls, replication, and sample sizes. The conclusions must be drawn appropriately based on the data presented.

Reviewer #3: Yes

Reviewer #4: Yes

Reviewer #5: Yes

**********

3. Has the statistical analysis been performed appropriately and rigorously?

Reviewer #3: Yes

Reviewer #4: Yes

Reviewer #5: Yes

**********

4. Have the authors made all data underlying the findings in their manuscript fully available?

The PLOS Data policy requires authors to make all data underlying the findings described in their manuscript fully available without restriction, with rare exception (please refer to the Data Availability Statement in the manuscript PDF file). The data should be provided as part of the manuscript or its supporting information, or deposited to a public repository. For example, in addition to summary statistics, the data points behind means, medians and variance measures should be available. If there are restrictions on publicly sharing data—e.g. participant privacy or use of data from a third party—those must be specified.

Reviewer #3: Yes

Reviewer #4: Yes

Reviewer #5: Yes

**********

5. Is the manuscript presented in an intelligible fashion and written in standard English?

PLOS ONE does not copyedit accepted manuscripts, so the language in submitted articles must be clear, correct, and unambiguous. Any typographical or grammatical errors should be corrected at revision, so please note any specific errors here.

Reviewer #3: Yes

Reviewer #4: Yes

Reviewer #5: Yes

**********

6. Review Comments to the Author

Please use the space provided to explain your answers to the questions above. You may also include additional comments for the author, including concerns about dual publication, research ethics, or publication ethics. (Please upload your review as an attachment if it exceeds 20,000 characters)

Reviewer #3: Comments are limited to editorial remarks and not the substance of the paper:

Figure 1 – Change “motorbike” to “motorcycle”

Line 228 – Delete “were” from this passage: “with 8.6% were on anti-epileptic medications” Should be “with 8.6% on anti-epileptic medications”

Line 240-241 – Replace “with” with “and”… “1.41 years and the majority were male (76.5%).”

Line 243 - Should be “accidents took place in the central region of Malaysia” Rather than “accident took place in central region of Malaysia”

Line 275 – Should read “Most of these accidents happened in the central region (47.8%), followed by the northern and east coast region” Rather than “Most of this accident happened at central region (47.8%), followed by northern and east coast region”

Line 293 – Should be “characteristics” rather than “characteristic”

Line 299 – Recommend “anti-epileptic drug prescription” rather than “anti-epileptic drug use”

Line 398 – “nationwide which include special education schools” Rather than “nationwide which includes the special education schools”

Line 421 – “A substantial proportion of children” Rather than “A substantial proportion of them”

Reviewer #4: Thanks for incorporating the suggestions. Limitations of study has been added by the authors in the revised version.

Reviewer #5: This study fills a regional data gap regarding outcomes of school aged children with RTA related TBI in Malaysia. The sample size is large, and the focus on academic difficulties after return to school is important but often overlooked. the paper has the potential to provide valuable references for TBI rehabilitation policy in Southeast Asia.

**********

7. PLOS authors have the option to publish the peer review history of their article (what does this mean?). If published, this will include your full peer review and any attached files.

If you choose “no”, your identity will remain anonymous but your review may still be made public.

**Do you want your identity to be public for this peer review?**   For information about this choice, including consent withdrawal, please see our Privacy Policy.

Reviewer #3: No

Reviewer #4: **Yes:**   Jamshed Akhtar

Reviewer #5: No

**********

# Editor report: Acceptance letter

**DOI:** 10.1371/journal.pone.0351136.r006

**Authors:** Nik Hisamuddin Nik Ab. Rahman

PONE-D-25-33135R2

PLOS One

Dear Dr. Mazlan,

I'm pleased to inform you that your manuscript has been deemed suitable for publication in PLOS One. Congratulations! Your manuscript is now being handed over to our production team.

At this stage, our production department will prepare your paper for publication. This includes ensuring the following:

* All references, tables, and figures are properly cited

* All relevant supporting information is included in the manuscript submission,

* There are no issues that prevent the paper from being properly typeset

You will receive further instructions from the production team, including instructions on how to review your proof when it is ready. Please keep in mind that we are working through a large volume of accepted articles, so please give us a few days to review your paper and let you know the next and final steps.

Lastly, if your institution or institutions have a press office, please let them know about your upcoming paper now to help maximize its impact. If they'll be preparing press materials, please inform our press team within the next 48 hours. Your manuscript will remain under strict press embargo until 2 pm Eastern Time on the date of publication. For more information, please contact onepress@plos.org.

You will receive an invoice from PLOS for your publication fee after your manuscript has reached the completed accept phase. If you receive an email requesting payment before acceptance or for any other service, this may be a phishing scheme. Learn how to identify phishing emails and protect your accounts at https://explore.plos.org/phishing.

If we can help with anything else, please email us at customercare@plos.org.

Thank you for submitting your work to PLOS ONE and supporting open access.

Kind regards,

PLOS ONE Editorial Office Staff

on behalf of

Professor Dr Nik Hisamuddin Nik Ab. Rahman

Academic Editor

PLOS One
